# Evaluating the Reliability of MyotonPro in Assessing Muscle Properties: A Systematic Review of Diagnostic Test Accuracy

**DOI:** 10.3390/medicina60060851

**Published:** 2024-05-23

**Authors:** Jonathan Lettner, Aleksandra Królikowska, Nikolai Ramadanov, Łukasz Oleksy, Hassan Tarek Hakam, Roland Becker, Robert Prill

**Affiliations:** 1 Center of Orthopaedics and Traumatology, University Hospital Brandenburg/Havel, Brandenburg Medical School, Hochstraße 29, 14770 Brandenburg an der Havel, Germany; jonathan.lettner@mhb-fontane.de (J.L.);; 2 Ergonomics and Biomedical Monitoring Laboratory, Department of Physiotherapy, Faculty of Health Sciences, Wroclaw Medical University, Tytusa Chalubinskiego 3, 50-368 Wroclaw, Poland; aleksandra.krolikowska@umw.edu.pl; 3 Faculty of Health Science, Brandenburg Medical School, 14770 Brandenburg an der Havel, Germany; 4 Department of Physiotherapy, Faculty of Health Sciences, Jagiellonian University Medical College, Michałowskiego 12, 31-126 Krakow, Poland; 5 Department of Orthopaedics, Traumatology and Hand Surgery, Faculty of Medicine, Wroclaw Medical University, Borowska 213, 50-556 Wroclaw, Poland

**Keywords:** MyotonPro, reliability, intra-rater reliability, inter-rater reliability, muscle properties

## Abstract

*Background and Objectives:* Muscle properties are critical for performance and injury risk, with changes occurring due to physical exertion, aging, and neurological conditions. The MyotonPro device offers a non-invasive method to comprehensively assess muscle biomechanical properties. This systematic review evaluates the reliability of MyotonPro across various muscles for diagnostic purposes. *Materials and Methods:* Following PRISMA guidelines, a comprehensive literature search was conducted in Medline (PubMed), Ovid (Med), Epistemonikos, Embase, Cochrane Library, Clinical trials.gov, and the WHO International Clinical Trials platform. Studies assessing the reliability of MyotonPro across different muscles were included. A methodological quality assessment was performed using established tools, and reviewers independently conducted data extraction. Statistical analysis involved summarizing intra-rater and inter-rater reliability measures across muscles. *Results:* A total of 48 studies assessing 31 muscles were included in the systematic review. The intra-rater and inter-rater reliability were consistently high for parameters such as frequency and stiffness in muscles of the lower and upper extremities, as well as other muscle groups. Despite methodological heterogeneity and limited data on specific parameters, MyotonPro demonstrated promising reliability for diagnostic purposes across diverse patient populations. *Conclusions:* The findings suggest the potential of MyotonPro in clinical assessments for accurate diagnosis, treatment planning, and monitoring of muscle properties. Further research is needed to address limitations and enhance the applicability of MyotonPro in clinical practice. Reliable muscle assessments are crucial for optimizing treatment outcomes and improving patient care in various healthcare settings.

## 1. Introduction

The properties of the muscles play a decisive role in terms of muscle performance [1]. Changes in these properties can be observed following physical exertion, such as impairment of speed, increases in stiffness after hopping, and the escalation of injuries associated with increasing muscle stiffness in tennis players [2,3,4,5,6,7,8]. High levels of muscle stiffness are not only associated with increased muscle performance, but also with an increased risk of muscle injury [2,9,10,11,12]. The most common muscle injuries in sport include muscle strains, muscle fiber tears, and muscle tears caused by sudden overstretching of tensed muscles, for example, during sprinting or sudden braking [13,14,15]. These injuries can also be caused by increased muscle stiffness [1,14,16,17]. It is important to note that muscle stiffness increases not only in response to muscular strain, but also with age [14]. Furthermore, changes in muscle properties can occur after paresis [18] and strokes [19].

Muscle tone can be assessed using non-instrumental methods, such as clinical scales, or instrumental tools, such as electrophysiological or mechanical measurements. Clinical scales are subjective assessment tools, while electrophysiological or mechanical measurements can provide objective information about muscle tone. In the clinical environment, cost-effective, user-friendly, and easy-to-use devices are required.

The MyotonPro (Myoton AS Tallinn, Estonia), introduces an innovative and non-invasive approach for comprehensively characterizing the biomechanical and viscoelastic properties of muscles. This advanced device allows for the assessment of various key parameters, including stiffness, tone (frequency), elasticity (decrement), relaxation time, and creep, providing a thorough analysis of muscle functionality [20,21]. With its new technology, the MyotonPro enables a precise and detailed understanding of the mechanical behavior of muscles, contributing valuable insights into their dynamic properties and potential functional implications.

The stiffness refers to the resistance of soft tissues to external forces and is calculated by analyzing damped natural vibrations detected by an integrated accelerometer. The quantification of muscle tone is derived from the natural frequency of the acceleration signal and provides valuable insight into the intrinsic properties of the muscle. In addition, muscle elasticity, which is inversely related to decrement, is assessed by analyzing the sequential oscillations that occur when the muscle returns to its original shape after deformation [22].

Tension relaxation time reflects the duration of the muscle’s recovery process [22,23]. It is a measure of the time required for the muscle to return to its initial state. On the other hand, muscle creep is defined as the gradual stretching of the muscle under a constant tensile stress [20,23]. This phenomenon provides crucial information about the structural properties of the muscle and its response to sustained loading and contributes to a comprehensive understanding of its viscoelastic behavior. The MyotonPro, through its advanced technology, enables a thorough investigation of these different parameters and provides a holistic perspective on the complex biomechanics and viscoelastic properties of muscles.

The reliability of measurement tools and devices is paramount in ensuring the validity and trustworthiness of results obtained from such measurements. Reliability pertains to the consistency and dependability of a measurement or assessment tool in producing similar results under consistent conditions. The reliability can be subdivided into intra-rater and inter-rater reliability. Both intra-rater and inter-rater reliability play crucial roles in assessing the accuracy of a measurement instrument. Intra-rater reliability involves a single rater, whereas inter-rater reliability necessitates the participation of at least two raters. It is worth noting that, particularly in clinical practice, inter-rater reliability holds greater significance as it more accurately reflects real-world application [24].

This comprehensive systematic review investigates the reliability of the MyotonPro as an assessment tool for various muscle groups, encompassing the upper and lower limbs and other muscles not confined to these regions. The review aims to offer a comprehensive understanding of the instrument’s applicability across diverse anatomical regions.

## 2. Materials and Methods

### 2.1. Protocol Design

For this systematic review, we followed the guidelines of the Preferred Reporting Items for Systematic Reviews and Meta-Analyses (PRISMA) [25,26]. The PRISMA guidelines are a recognized and standardized method for conducting systematic reviews and meta-analyses in research [27]. Compliance with these guidelines ensures a transparent and structured approach to conducting and reporting systematic reviews. This study has been registered in PROSPERO under registration number: CRD42024497053.

### 2.2. Search Strategy

A literature review was conducted in Medline (PubMed), Ovid (Med), Epistemonikos, Embase, Cochrane Library, Clinical trials.gov, and the WHO International Clinical Trials platform until 31 January 2023. The following search terms were used:

((((((“MyotonPro” OR(“stiff” OR “stiffness” OR “stiffnesses”)) OR (“Muscle” AND “tone”) OR (“decrement” OR “decrements”)) AND (“reliabilities”nOR “reliability” OR “reliable” OR “reliability” OR “reliably”)) OR (“muscle s” OR “muscles” [MeSH Terms] OR “muscles” OR “muscle”) OR “Myoton” OR “Myotonometer”) OR (Relaxation)) (musculoskeletal disease)) OR (((Orthopedic*) OR (Physiotherapy)) OR (validity))

The resulting records from the search underwent title and abstract screening. We have not included studies published before June 2003.

### 2.3. Study Selection (Inclusion and Exclusion)

Articles were imported into JBI SUMARI (Joanna Briggs Institute) and duplicates were removed. Two reviewers (JL and PL) reviewed titles and abstracts for eligibility against predetermined criteria. The full-text articles were independently screened by two reviewers (JL and RP), the studies had to meet the eligibility criteria presented in Table 1.

The exclusion criteria included studies that either lacked essential reliability assessments or did not report their test results in the Intraclass Correlation Coefficient (ICC). Furthermore, studies focusing on reliability assessments using artificial tissue models were excluded from consideration. Where necessary, the authors were contacted for additional information to obtain complete information. If the authors could not be reached, the article was excluded from the review.

### 2.4. Methodological Quality Assessment

The methodological quality of each study was independently assessed by 2 reviewers (JL and RP) using the Cochrane risk-of-bias (ROB-2) tool of the second version for randomized controlled trials (RCTs) [28]. The inter-rater reliability of this tool has been shown to be fair to substantial depending on the assessment areas [28]. For non RCT-studies, we used the JBI tool for diagnostic test accuracy [29]. Any disagreements between reviewers were resolved through in-depth discussions. The use of this methodological approach helps to ensure the reliability and accuracy of the quality assessment and thus strengthens the reliability of the results of this review.

### 2.5. Data Extraction

The data were extracted by one reviewer (JL) in relation to sample size, population, intervention, setting, outcome measures, data collection time points, and results. The results were expressed in ICC. In cases where the information contained in the published articles or Supplementary Materials was not sufficient for a comprehensive understanding, attempts were made to contact the respective authors.

### 2.6. Statistical Analysis and Data Synthesis

All studies were entered into an Excel spreadsheet. Since many studies tested the reliability not only for 1 muscle but for up to 8 muscles, a second Excel sheet was created to sort the studies by muscles. In total, 31 different muscles and tendons from 48 studies were examined, with 11 belonging to the lower extremities, 9 to the upper extremities, and 11 to other anatomical region. The individual ICC results of each study for each muscle were then summed and divided by the number of studies using the weighted mean average. This process generated a new ICC value that incorporated all studies related to the specific muscle.

Subgroup analyses were undertaken to comprehensively explore potential variations across distinct muscle groups. This meticulous examination aimed to delve into the nuanced aspects of reliability within specific categories, such as muscles of the lower extremities, upper extremities, and other groups. By conducting subgroup analyses, we aimed to discern any unique patterns, trends, or specific considerations that may influence reliability outcomes within each muscle group. This approach not only enhances the granularity of our assessment but also allowed for a more nuanced understanding of the MyotonPro performance across an inverse range of anatomical regions.

We utilized the following categorization scheme to interpret the Intraclass Correlation Coefficient (ICC): poor reliability (<0.49), moderate reliability (0.5–0.75), good reliability (0.75–0.9), and excellent reliability (>0.91) [30].

## 3. Results

### 3.1. Results of Study Selection

**Figure 1:**
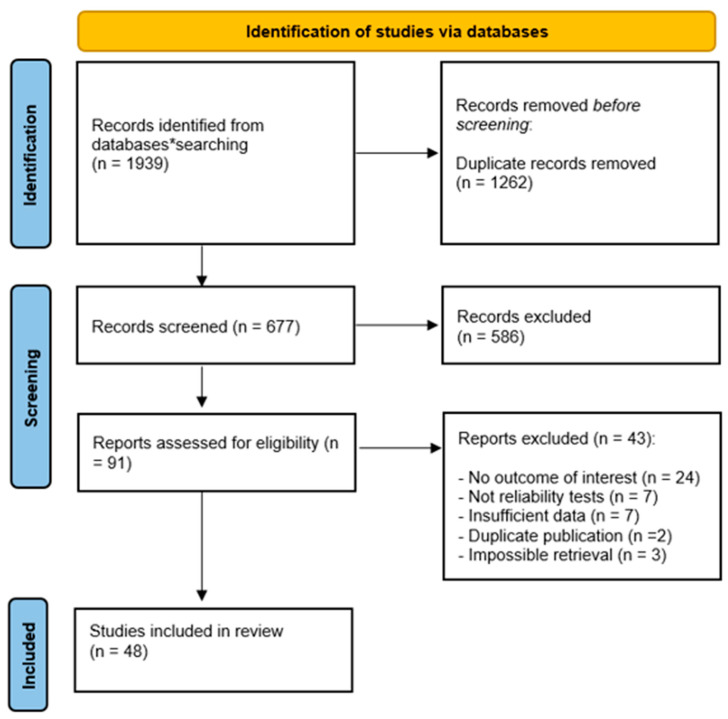
Presentation of the PRISMA statement.

All search records were initially compiled using EndNote (version 20, Clarivate Analytics, Philadelphia, PA, USA) and JBI SUMARI. A total of 1939 studies were identified based on the predefined search criteria. After an initial screening phase, 1262 datasets were excluded due to non-compliance with the inclusion criteria. Subsequently, 677 records underwent a dual screening process conducted by two independent reviewers (JL and RP). Out of these, 586 records were further excluded, leaving 91 records for more comprehensive examination. Ultimately, 43 studies were excluded for various reasons, including lack of outcomes of interest (*n* = 24), absence of reliability tests (*n* = 7), insufficient data (*n* = 7), duplicate publication (*n* = 2), and inability to retrieve the publication (*n* = 3). This resulted in the inclusion of 48 studies in the systematic review, achieving complete inter-reviewer agreement (k = 1.0). The detailed study selection process is illustrated in Figure 1.

The distribution of participants across various health conditions in the records is presented in Table 2. The included studies showed a distribution of the 1673 participants across 54 groups. The 1060 healthy participants were examined in 33 groups, thus significantly outnumbering the majority of the investigated groups.

The extensive findings, segmented according to different muscles and ligaments, are presented in two distinct tables provided within the Supplementary Materials. Table S1 in the Supplementary Material depicts the inter-rater reliability, while Table S2 showcases the intra-rater reliability.

### 3.2. Lower Limb

#### 3.2.1. Rectus Femoris Muscle

The diagnostic value of the rectus femoris muscle was evaluated in eleven studies [31,32,33,34,35,36,37,38,39,40,41]. The parameters frequency, stiffness, decrement, creep, and relaxation were recorded.

Frequency: Intra-rater reliability was investigated in four studies with a total of 86 healthy subjects [31,32,33,34]. The subjects were aged between 11 and 82 years. The reliability ranged between ICC 0.63 and ICC 0.99 and came to a weighted mean average of ICC 0.89. In addition, a total of 28 patients with stroke were examined in one study [35]. They achieved an ICC value of 0.81. Another study examined 15 children aged 11 with cerebral palsy [31]. They achieved an ICC value of 0.84. A total of 13 male patients with spinal cord injuries were also examined in one study [36]. They achieved an ICC value of 0.96.

The inter-rater reliability was examined in six studies with a total of 185 healthy test subjects [32,33,34,37,38,39]. The subjects were aged between 20 and 82 years. The reliability ranged between 0.63 and 0.97, with a weighted mean average ICC of 0.85. In addition, a total of 48 patients with stroke were examined in two studies [37,40]. They were aged between 30 and 83 years, and 14 were female and 34 were male. They achieved an ICC value of 0.75 to 0.86 and thus a weighted mean average of 0.81. In addition, 15 children aged 11 with cerebral palsy were examined [31]. These children had an ICC value of 0.68.

Stiffness: Intra-rater reliability was examined in four studies with a total of 111 healthy test subjects [32,33,34,41]. The subjects were aged between 19 and 82 years, and 35 were female and 76 were male. The reliability ranged between ICC 0.78 and ICC 0.99 and came to a weighted mean average of ICC 0.91. In addition, 13 male patients with spinal cord injuries were examined in one study [36]. These patients achieved an ICC value of 0.96.

The inter-rater reliability was examined in six studies with a total of 185 healthy test subjects [32,33,34,37,38,39]. The subjects were aged between 20 and 82 years. The reliability ranged between ICC 0.82 and ICC 0.97, with a weighted mean average ICC of 0.90. In addition, a total of 48 patients with stroke were examined in two studies [37,40]. They were aged between 30 and 83 years, and 14 were female and 34 were male. They achieved an ICC value of 0.87 to 0.99 and thus a weighted mean average ICC of 0.93.

Decrement: Intra-rater reliability was examined in two studies with a total of 41 healthy male subjects [32,33]. The subjects were aged between 20 and 82 years. The reliability in both studies was ICC 0.99. In addition, 13 male patients with spinal cord injuries were examined in one study [36]. These patients had an ICC value of 0.68.

The inter-rater reliability was examined in five studies with a total of 155 healthy test subjects [32,33,37,38,39]. The subjects were aged between 20 and 82 years. The reliability ranged between ICC 0.77 and ICC 0.88, with a weighted mean average ICC of 0.82. In addition, a total of 48 patients with stroke were examined in two studies [37,40]. They were aged between 30 and 83 years, and 14 were female and 34 were male. They achieved an ICC value of 0.77 to 0.81 and thus a weighted mean average ICC of 0.79.

Relaxation: The intra-rater reliability was examined in a study with 13 male spinal cord injury patients [36]. They achieved an ICC value of 0.99.

The inter-rater reliability was not assessed.

Creep: The intra-rater reliability was examined in a study with 13 male spinal cord injury patients [36]. They arrived at an ICC value of 0.98.

The inter-rater reliability was examined in a study with 29 stroke patients [40]. They were between 30 and 83 years old, with 5 females and 24 males. They achieved an ICC value of 0.92.

#### 3.2.2. Vastus Lateralis Muscle

The diagnostic value of the vastus lateralis muscle was evaluated in four studies [34,37,39,42]. The parameters frequency, stiffness, and decrement were recorded.

Frequency: Intra-rater reliability was examined in one study with a total of 30 healthy test subjects [34]. The subjects were aged between 23 and 27 years, with 15 females and 15 males. The reliability ICC ranged between 0.65 and 0.82, resulting in a weighted mean average ICC of 0.74.

The inter-rater reliability was examined in three studies with a total of 102 healthy test subjects [34,37,39]. The subjects were aged between 23 and 63 years. The reliability ICC ranged between 0.91 and 0.98, resulting in a weighted mean average ICC of 0.95. In addition, 20 patients with stroke were examined in one study [37]. The patients were aged between 43 and 63 years, with 9 females and 11 males. They had an ICC value of 0.91.

Stiffness: Intra-rater reliability was examined in two studies with a total of 83 healthy test subjects [37,42]. The subjects were aged between 19 and 37 years, and 35 were female and 47 were male. The reliability ranged between 0.65 and 0.97, resulting in a weighted mean average ICC of 0.81.

The inter-rater reliability was examined in four studies with a total of 155 healthy test subjects [34,37,39,42]. The subjects were aged between 19 and 63 years. The reliability ranged between ICC 0.91 and ICC 0.96, resulting in a mean value of 0.94. In addition, 20 patients with stroke were examined in one study [37]. The patients were aged between 43 and 63 years, with 9 females and 11 males. They had an ICC value of 0.92.

Decrement: Intra-rater reliability was not investigated in any study.

The inter-rater reliability was examined in two studies with a total of 72 healthy test subjects [37,39]. The reliability ranged between ICC 0.83 and ICC 0.91, resulting in a weighted mean average ICC of 0.87. In addition, 20 patients with stroke were examined in one study [37]. The patients were aged between 43 and 63 years, with 9 females and 11 males. They had an ICC value of 0.83.

#### 3.2.3. Vastus Medialis Muscle

The diagnostic value of the vastus medialis muscle was evaluated in one study [34]. The parameters frequency and stiffness were recorded.

Frequency: The intra-rater reliability was examined in one study with 30 healthy test subjects [34]. Thy aged 23 to 27 years, 15 of whom were women and 15 men. The reliability ranged between ICC 0.79 and ICC 0.80, resulting in a weighted mean average ICC of 0.80.

The inter-rater reliability of the same study with the same test subjects achieved a reliability of ICC 0.96 to ICC 0.91, resulting in a weighted mean average ICC of 0.94.

Stiffness: The intra-rater reliability of the same study was ICC 0.62 to ICC 0.79, resulting in a weighted mean average ICC of 0.71. The inter-rater reliability was ICC 0.91 and ICC 0.96, resulting in a weighted mean average ICC of 0.94.

#### 3.2.4. Biceps Femoris Muscle

Four studies [41,43,44,45] evaluated the diagnostic value of the biceps femoris muscle. The parameters frequency, stiffness, decrement, creep, and relaxation were recorded.

Frequency: Intra-rater reliability was examined in one study with 21 healthy test subjects [43]. The subjects were aged between 20 and 35 years, and only men were examined. The reliability was an ICC value of 0.99. In addition, 13 male patients with spinal cord injuries were examined in a study [44]. This study had an ICC value of 0.92.

The inter-rater reliability was examined in two studies with 41 healthy test subjects [43,45]. The subjects were aged between 20 and 63 years, 32 men and 9 women were examined. The reliability ranged between ICC 0.75 and ICC 0.86, resulting in a weighted mean average ICC of 0.81. In addition, 20 patients with stroke were examined in one study [45]. The patients were aged between 43 and 63 years, and 9 were female and 11 were male. They had an ICC value of 0.75.

Stiffness: Intra-rater reliability was examined in two studies with a total of 61 healthy test subjects [41,43]. The subjects were aged between 19 and 35 years, and 20 were female and 41 were male. The reliability ranged between ICC 0.86 and ICC 0.99, resulting in a weighted mean average ICC of 0.91. In addition, 13 male patients with spinal cord injuries were examined in one study [44]. These patients achieved a weighted mean average ICC of 0.95.

The inter-rater reliability was examined in two studies with a total of 41 healthy test subjects [43,45]. The subjects were aged between 20 and 63 years, and 9 were female and 32 were male. The reliability ranged between ICC 0.72 and ICC 0.80, resulting in a weighted mean average ICC of 0.76. In addition, a total of 20 patients with stroke were examined in one study [45]. The patients were aged between 43 and 63 years, and 9 were female and 11 were male. They achieved an ICC value of 0.80.

Decrement: Intra-rater reliability was examined in one study with 21 healthy test subjects [43]. The subjects were aged between 20 and 35 years and only men were examined. The reliability was 0.99. In addition, 13 exclusively male patients with spinal cord injuries were examined in one study [44]. These patients had an ICC value of 0.82.

The inter-rater reliability was examined in two studies with a total of 41 healthy test subjects [43,45]. The subjects were aged between 20 and 63 years, and 9 were female and 32 were male. The reliability ranged between ICC 0.77 and ICC 0.78, resulting in a weighted mean average ICC of 0.78. In addition, 20 patients with stroke were examined in one study [45]. The patients were aged between 43 and 63 years, and 9 were female and 11 were male. They had an ICC value of 0.78.

Relaxation: Intra-rater reliability was only performed in one study with 13 male spinal cord injections patients [44]. The ICC value was 0.96. No study with healthy volunteers has been conducted to date.

No study on inter-rater reliability has been conducted to date.

Creep: Intra-rater reliability was only performed in one study with 13 male spinal cord injections patients [44]. The ICC value was 0.96. No study with healthy volunteers has been conducted to date.

No study on inter-rater reliability has been conducted to date.

#### 3.2.5. Patellar Ligament

Four studies [34,39,46,47] assessed the diagnostic value of patellar ligament. The parameters frequency, stiffness, and decrement were recorded. In addition, only healthy test subjects were examined.

Frequency: Intra-rater reliability was examined in three studies with a total of 60 healthy test subjects [34,46,47]. The subjects were aged between 20 and 28 years, and 25 were female and 35 were male. The reliability ranged between ICC 0.68 and ICC 0.96, resulting in a weighted mean average ICC of 0.79.

The inter-rater reliability was examined in three studies with a total of 102 healthy test subjects [34,39,47]. The test subjects were under 30 years of age. The reliability ranged between ICC 0.85 and ICC 0.95 and came to a weighted mean average ICC of 0.91.

Stiffness: Intra-rater reliability was examined in three studies with a total of 60 healthy test subjects [34,46,47]. The subjects were aged between 20 and 28 years, and 25 were female and 35 were male. The reliability ranged between ICC 0.68 and ICC 0.96, with a mean value of 0.81.

The inter-rater reliability was examined in three studies with a total of 102 healthy test subjects [34,39,47]. The subjects were under 30 years of age. The reliability ranged between 0.85 and 0.95 and came to a weighted mean average ICC of 0.92.

Decrement: Intra-rater reliability was investigated in two studies with a total of 30 healthy volunteers [46,47]. The test subjects were all under 30 years of age. The reliability ranged between ICC 0.68 and ICC 0.86, with a weighted mean average ICC of 0.77.

The inter-rater reliability was examined in two studies with a total of 72 healthy test subjects [39,47]. The test subjects were under 30 years of age. The reliability ranged between ICC 0.85 and ICC 0.95, with a weighted mean average ICC of 0.90.

#### 3.2.6. Gastrocnemius Medialis Muscle

The diagnostic value of the gastrocnemius medialis muscle was evaluated in eight studies [36,37,41,48,49,50,51,52]. The parameters frequency, stiffness, decrement, creep, and relaxation were recorded.

Frequency: The intra-rater reliability was examined in one study with 13 male patients with spinal cord injuries [36]. These achieved an ICC value of 0.91. No study with healthy subjects has yet been undertaken.

The inter-rater reliability was investigated in one study with a total of 20 healthy volunteers [37]. The subjects were aged between 43 and 63 years, and 9 were female and 11 were male. The reliability was ICC 0.69. In addition, 20 patients with stroke were examined in the same study. These also had an ICC value of 0.69.

Stiffness: Intra-rater reliability was examined in four studies with a total of 100 healthy test subjects [41,48,49,50]. The subjects were aged between 19 and 63 years. The reliability ranged between ICC 0.78 and ICC 0.99, with a weighted mean average ICC of 0.87. In addition, a total of 27 patients with spinal cord injuries were examined in two studies [36,51]. These achieved an ICC value of 0.87 to 0.97 and thus a weighted mean average ICC of 0.91.

The inter-rater reliability was examined in two studies with a total of 39 healthy subjects [37,52]. The reliability ranged between ICC 0.77 and ICC 0.95 and came to a weighted mean average ICC of 0.87. In addition, 20 patients with stroke were examined in one study [37]. The subjects were aged between 43 and 63 years, and 9 were female and 11 were male. The reliability was 0.77. In addition, 13 male patients with spinal cord injuries were examined in one study [51]. These also achieved an ICC value of 0.98.

Decrement: The intra-rater reliability was examined exclusively in one study with 13 male patients suffering from spinal cord injuria [36]. The ICC value was 0.81.

The inter-rater reliability was examined exclusively in one study with 20 patients suffering from spinal cord injuria [51]. Age was between 43 and 63 years, and 9 were female and 11 were male. The reliability was ICC 0.62.

No study with healthy volunteers has been undertaken to date.

Relaxation: The intra-rater reliability was examined exclusively in one study with 13 male patients suffering from spinal cord injuria [36]. The ICC value was 0.89.

An inter-rater reliability study has not yet been conducted. Furthermore, no study with healthy volunteers has been undertaken.

Creep: The intra-rater reliability was examined exclusively in one study with 13 male patients suffering from spinal cord injuria [51]. The ICC value was 0.89.

An inter-rater reliability study has not yet been conducted. Furthermore, no study with healthy volunteers has been undertaken.

#### 3.2.7. Gastrocnemius Lateralis Muscle

The diagnostic value of the gastrocnemius lateralis muscle was evaluated in six studies [36,43,49,50,51,53]. The parameters frequency, stiffness, decrement, creep, and relaxation were recorded.

Frequency: The intra-rater reliability was examined exclusively in one study with 13 male patients suffering from spinal cord injuria [36]. The ICC value was 0.64.

An inter-rater reliability study has not yet been conducted. Furthermore, no study with healthy volunteers has been undertaken.

Stiffness: Intra-rater reliability was investigated in four studies with a total of 149 healthy volunteers [43,49,50,53]. The subjects were aged between 21 and 42 years. The reliability ranged between ICC 0.84 and ICC 0.99, with a weighted mean average ICC of 0.92. In addition, a total of 27 patients with spinal cord injuries were examined in two studies [36,51]. Of the patients, 2 were female and 25 were male. The reliability ranged between ICC 0.91 and ICC 0.95, with a weighted mean average ICC of 0.93.

The inter-rater reliability was examined in one study with 35 healthy test subjects [43]. The subjects were aged between 22 and 42 years. The reliability was ICC 0.86. In addition, 14 patients with spinal cord injuries were examined in one study [51]. Of the patients, 2 were female and 12 were male. The reliability was ICC 0.98.

Decrement: The intra-rater reliability was examined exclusively in one study with 13 male patients suffering from spinal cord injuria [36]. The ICC value was 0.79.

An inter-rater reliability study has not yet been conducted. Furthermore, no study with healthy volunteers has been undertaken.

Relaxation: The intra-rater reliability was examined exclusively in a study with 13 male patients suffering from spinal cord injuria [36]. The ICC value was 0.86.

An inter-rater reliability study has not yet been conducted. Furthermore, no study with healthy volunteers has been undertaken.

Creep: Intra-rater reliability was examined exclusively in a study with 13 male patients suffering from spinal cord injuria [36]. The ICC value was 0.86.

An inter-rater reliability study has not yet been conducted. Furthermore, no study with healthy subjects has been undertaken.

#### 3.2.8. Soleus Muscle

Two studies [47,54] assessed the diagnostic value of soleus muscle. The parameters frequency, stiffness, and decrement were recorded. In addition, only healthy test subjects were examined.

Frequency: Intra-rater reliability was investigated in one study with 20 healthy volunteers [47]. The test subjects were 10 females and 10 males. The reliability was ICC 0.90.

The inter-rater reliability was examined in two studies with a total of 70 healthy test subjects [47,54]. The subjects were aged between 17 and 33 years, and 30 were female and 40 were male. The reliability ranged between ICC 0.87 and ICC 0.97, with a weighted mean average ICC of 0.93.

Stiffness: Intra-rater reliability was investigated in a study with 20 healthy volunteers [47]. The subjects were 10 females and 10 males. The reliability was ICC 0.66.

Inter-rater reliability was investigated in two studies with a total of 70 healthy volunteers [47,54]. The test subjects were aged between 17 and 33 years, and 30 were female and 40 were male. The reliability ranged between ICC 0.93 and ICC 0.95, with a weighted mean average ICC of 0.94.

Decrement: Intra-rater reliability was investigated in a study with 20 healthy volunteers [47]. The subjects were 10 females and 10 males. The reliability was ICC 0.71.

The inter-rater reliability was examined in two studies with a total of 70 healthy test subjects [47,54]. The subjects were aged between 17 and 33 years, and 30 were female and 40 male. The reliability ranged between 0.65 and 0.94, with a mean value of 0.817.

#### 3.2.9. Tibialis Anterior Muscle

Six studies [35,37,40,41,47,55] evaluated the diagnostic value of the tibialis anterior muscle. The parameters frequency, stiffness, decrement, and creep were recorded.

Frequency: Intra-rater reliability was examined in one study with 20 healthy test subjects [47]. Of the test subjects, 10 were female and 10 were male. The reliability was 0.80. In addition, 28 patients with stroke were examined in a study [35]. These achieved an ICC value of 0.81. A further study examined 30 patients with Parkinson’s disease [55]. The reliability here was 0.94.

The inter-rater reliability was examined in two studies with a total of 40 healthy test subjects [37,47]. Of the test subjects, 19 were female and 21 were male. The reliability ranged between ICC 0.76 and ICC 0.91, with a weighted mean average ICC of 0.84. In addition, a total of 49 patients with stroke were examined in two studies [37,40]. The patients were aged between 30 and 83 years, and 14 were female and 34 were male. The reliability ranged between ICC 0.76 and ICC 0.91, with a weighted mean average ICC of 0.85.

Stiffness: Intra-rater reliability was investigated in two studies with 60 healthy volunteers [41,47]. Of the test subjects, 30 were female and 30 were male. The ICC reliability ranged between 0.85 and 0.98 and came to a weighted mean average ICC of 0.87. In addition, 30 patients with Parkinson’s disease were examined in one study [55]. These patients achieved an ICC value of 0.97.

The inter-rater reliability was examined in two studies with a total of 40 healthy test subjects [37,47]. Of the test subjects, 19 were female and 21 were male. The reliability ranged between 0.79 and 0.93, with a weighted mean average ICC of 0.86. In addition, a total of 49 patients with stroke were examined in two studies [37,40]. The patients were aged between 30 and 83 years, and 14 were female and 34 were male. The ICC reliability ranged between 0.79 and 0.91, with a weighted mean average ICC of 0.84.

Decrement: Intra-rater reliability was examined in one study with 20 healthy test subjects [47]. Of the subjects, 10 were female and 10 were male. The reliability was ICC 0.95. In addition, 30 patients with Parkinson’s disease were examined in one study [55]. These had an ICC value of 0.95.

The inter-rater reliability was examined in two studies with a total of 40 healthy test subjects [41,47]. Of the test subjects, 19 were female and 21 were male. The reliability ranged between ICC 0.71 and ICC 0.87, with a weighted mean average ICC of 0.79. In addition, a total of 49 patients with stroke were examined in two studies [37,40]. The patients were aged between 30 and 83 years, and 14 were female and 34 were male. The reliability ranged between ICC 0.65 and ICC 0.83, with a weighted mean average ICC of 0.73.

Creep: Only the inter-rater reliability was assessed in a study with 29 stroke patients [40]. The patients were aged between 30 and 83 years, and 5 were female and 24 were male. The reliability was ICC 0.90.

No studies on intra-rater reliability or studies with healthy subjects have been conducted to date.

#### 3.2.10. Achilles Tendon

Ten studies [36,42,46,47,49,50,52,56,57,58] evaluated the diagnostic value of Achilles tendon. The parameters frequency, stiffness, decrement, creep, and relaxation were assessed.

Frequency: Intra-rater reliability was examined in two studies with a total of 30 healthy test subjects [42,47]. The subjects were aged between 20 and 28 years, and 10 were female and 20 were male. The reliability ranged between 0.77 and 0.96 and came to a weighted mean average ICC of 0.87. In addition, 13 exclusively male patients with spinal cord injuries were examined in one study [36]. These patients achieved an ICC value of 0.82.

The inter-rater reliability was only examined in one study with 20 healthy test subjects [47]. There were 10 females and 10 males. The reliability was ICC 0.88.

Stiffness: Intra-rater reliability was examined in seven studies with a total of 150 healthy subjects [36,46,47,49,50,56,57]. The reliability ranged between ICC 0.74 and ICC 0.96 with a weighted mean average ICC of 0.80. In addition, 27 patients with spinal cord injuries were examined in two studies [36,51]. The reliability ranged between ICC 0.81 and ICC 0.89, with a weighted mean average ICC of 0.85.

The inter-rater reliability was examined in five studies with a total of 139 healthy test subjects [47,52,56,57,58]. The reliability ranged between ICC 0.87 and ICC 0.95 and came to a weighted mean average ICC of 0.92. In addition, 14 patients with spinal cord injuries were examined in one study [51]. There were 2 females and 12 males. They achieved an ICC value of 0.98.

Decrement: Intra-rater reliability was investigated in two studies with a total of 30 healthy volunteers [46,47]. There were 10 females and 20 males. The reliability ranged between ICC 0.68 and ICC 0.94 and came to a weighted mean average ICC of 0.81. In addition, 13 exclusively male patients with spinal cord injuries were examined in one study [36]. These patients achieved an ICC value of 0.58.

The inter-rater reliability was examined in a study with 20 healthy test subjects [47]. There were 10 females and 10 males. The reliability was ICC 0.85.

Relaxation: Only the intra-rater reliability was examined in a study with 13 male patients with spinal cord injuries [36]. The reliability was ICC 0.82.

Creep: Only the intra-rater reliability was examined in a study with 13 male patients with spinal cord injuries [36]. These patients achieved an ICC value of 0.89.

#### 3.2.11. Plantar Fascia

One study [47] assessed the diagnostic value of plantar fascia. The parameters frequency, stiffness, and decrement were recorded. In addition, only healthy test subjects were examined.

Frequency: Intra-rater reliability was examined in a study with a total of 20 healthy test subjects [47]. There were 10 females and 10 males. These patients achieved an ICC value of 0.77.

The inter-rater reliability was examined in the same study with the same 20 healthy test subjects. The reliability was 0.92.

Stiffness: Intra-rater reliability was examined in a study with a total of 20 healthy test subjects [47]. There were 10 females and 10 males. These patients achieved an ICC value of 0.96.

The inter-rater reliability was examined in the same study with the same 20 healthy test subjects. The ICC value wa0.97.

Decrement: Intra-rater reliability was examined in a study with a total of 20 healthy test subjects [47]. There were 10 females and 10 males. These patients achieved an ICC value of 0.55.

The inter-rater reliability was examined in the same study with the same 20 healthy test subjects. The reliability was ICC 0.90.

### 3.3. Upper Limb

#### 3.3.1. Infraspinatus Muscle

Two studies [48,59] evaluated the diagnostic value of the infraspinatus muscle. The parameters frequency, stiffness, and decrement were recorded.

Frequency: Intra-rater reliability was not investigated in any study with healthy subjects. It was only examined in a study with 35 patients with shoulder pain [59]. The patients were 42 years old, and 23 were female and 12 were male. The ICC value was 0.97.

The same applies to inter-rater reliability, which was also only examined in the aforementioned study. The ICC value here was 0.91.

Stiffness: Intra-rater reliability was examined in a study with a total of 30 healthy test subjects [48]. The reliability was ICC 0.98. In addition, 35 patients with shoulder pain were examined in a study [59]. The patients were 42 years old, and 23 were female and 12 were male. The ICC value was 0.96.

The same applies to inter-rater reliability, which was also only examined in the aforementioned study with shoulder pain patients. The ICC value here was 0.88.

Decrement: Intra-rater reliability was not investigated in any study with healthy subjects. It was only examined in one study with 35 patients with shoulder pain [59]. The patients were 42 years old, and 23 were female and 12 were male. The ICC value was 0.96.

The same applies to inter-rater reliability, which was also only examined in the aforementioned study. The ICC value here was 0.91.

#### 3.3.2. Deltoideus Muscle

Two studies [45,47] assessed the diagnostic value of deltoid muscle. The parameters frequency, stiffness, and decrement were recorded.

Frequency: Intra-rater reliability was examined in a study with a total of 20 healthy subjects [47]. There were 10 females and 10 males. The reliability was ICC 0.75. In addition, a total of 61 patients with stroke were examined in one study [45]. The patients were aged between 44 and 66 years, and 25 were female and 36 were male. They achieved an ICC value of 0.92.

The inter-rater reliability was examined in the same study with the same 20 healthy subjects [47]. The reliability was 0.80.

Stiffness: The intra-rater reliability was examined in a study with a total of 20 healthy test subjects [47]. There were 10 females and 10 males. The reliability was ICC 0.92. In addition, a total of 61 patients with stroke were examined in one study [45]. The patients were aged between 44 and 66 years, with 25 females and 36 males. They had an ICC value of 0.93.

The inter-rater reliability was examined in the same study with the same 20 healthy subjects [47]. The reliability was ICC 0.89.

Decrement: The intra-rater reliability was examined in a study with a total of 20 healthy test subjects [47]. There were 10 females and 10 males. The reliability was 0.94. In addition, a total of 61 patients with stroke were examined in one study [45]. The patients were aged between 44 and 66 years, with 25 females and 36 males. They had an ICC value of 0.86.

The inter-rater reliability was examined in the same study with the same 20 healthy subjects [47]. The reliability was 0.77.

#### 3.3.3. Biceps Brachii Muscle

The diagnostic value of the biceps brachii muscle was evaluated in eleven studies [35,38,40,43,44,45,55,60,61,62]. The parameters evaluated were frequency, stiffness, decrement, and creep.

Frequency: Intra-rater reliability was examined in a study with a total of 38 healthy test subjects [60]. The subjects were aged between 24 and 81 years, and 27 were female and 11 were male. The reliability was ICC 0.93. In addition, a total of 101 patients with stroke were examined in three studies [35,44,45]. They were aged between 40 and 66 years. They achieved an ICC value of 0.72 to 0.98 and thus a weighted mean average ICC of 0.83. In addition, a total of 86 patients with paratonia were examined in two studies [60,61]. They were aged between 70 and 98 years. They achieved an ICC value of 0.23 to 0.60 and thus a weighted mean average ICC of 0.47. A total of 30 patients with Parkinson’s disease were also examined in one study [55]. They achieved an ICC value of 0.94.

The inter-rater reliability was examined in two studies with a total of 80 healthy test subjects [38,60]. The subjects were aged between 20 and 81 years, and 48 were female and 32 were male. The reliability ranged between ICC 0.67 and ICC 0.84, with a weighted mean average ICC of 0.76. In addition, a total of 86 patients with paratonia were examined in two studies [60,61]. They were aged between 70 and 98 years. They achieved an ICC value of 0.43 to 0.70 and thus a weighted mean average ICC of 0.57. In addition, 29 patients with stroke were examined in one study [40]. These were aged between 30 and 83 years. There were 24 males and 5 females. They had an ICC value of 0.77.

Stiffness: Intra-rater reliability was examined in three studies with a total of 103 healthy test subjects [43,60,62]. The subjects were aged between 22 and 81 years. The reliability ranged between ICC 0.39 and ICC 0.99, with a weighted mean average ICC of 0.85. In addition, a total of 73 patients with stroke were examined in two studies [44,45]. They were aged between 40 and 66 years. They achieved an ICC value of 0.87 to 0.92 and thus a weighted mean average ICC of 0.0.90. In addition, a total of 86 patients with paratonia were examined in two studies [60,61]. They were aged between 70 and 98 years. They achieved an ICC value of 0.59 to 0.63 and thus a weighted mean average ICC of 0.61. A total of 38 patients with Parkinson’s disease were also examined in two studies [55,62]. They achieved an ICC value of 0.97 to 0.98 and thus a weighted mean average ICC of 0.98.

The inter-rater reliability was examined in four studies with a total of 126 healthy test subjects [38,43,60,63]. The subjects were aged between 20 and 81 years. The reliability ranged between ICC 0.75 and ICC 0.96, with a weighted mean average ICC of 0.85. In addition, a total of 86 patients with paratonia were examined in two studies [60,61]. They were aged between 70 and 98 years. They achieved an ICC value of 0.73 to 0.75 and thus a weighted mean average ICC of 0.72. A total of 29 patients with stroke were also examined in one study [40]. They were aged between 44 and 66 years. There were 24 males and 5 females. They had an ICC value of 0.71.

Decrement: Intra-rater reliability was examined in a study with a total of 38 healthy test subjects [62]. The subjects were aged between 24 and 81 years, and 27 were female and 11 were male. The reliability was ICC 0.74. In addition, a total of 73 patients with stroke were examined in two studies [44,45]. They were aged between 40 and 66 years. They achieved an ICC value of 0.90 to 0.93 and thus a weighted mean average ICC of 0.915. In addition, a total of 86 patients with paratonia were examined in two studies [60,61]. They were aged between 70 and 98 years. They achieved an ICC value of 0.35 to 0.88 and thus a weighted mean average ICC of 0.67. A total of 30 patients with Parkinson’s disease were also examined in one study [55]. They achieved an ICC value of 0.95.

The inter-rater reliability was examined in two studies with a total of 80 healthy test subjects [38,60]. The subjects were aged between 20 and 81 years, and 48 were female and 32 were male. The reliability ranged between ICC 0.78 and ICC 0.90, with a weighted mean average ICC of 0.87. In addition, a total of 86 patients with paratonia were examined in two studies [60,61]. They were aged between 70 and 98 years. They achieved an ICC value of 0.62 to 0.64 and thus a weighted mean average ICC of 0.63. In addition, 29 patients with stroke were examined in one study [40]. These were aged between 30 and 83 years. There were 24 males and 5 females. They achieved an ICC value of 0.82.

Creep: Intra-rater reliability was examined exclusively in a study with a total of 70 patients with paratonia [61]. This came to an ICC value of 0.54.

The inter-rater reliability was examined exclusively in a study with a total of 70 patients with paratonia [61]. This achieved an ICC value of 0.57. In addition, 29 patients with stroke were examined in one study [40]. These were aged between 30–83 years. There were 24 males and 5 females. They achieved an ICC value of 0.76.

#### 3.3.4. Triceps Brachii Muscle

Three studies [44,45,62] assessed the diagnostic value of triceps brachii muscle. The parameters frequency, stiffness, and decrement were assessed. Inter-rater reliability was not examined in any study.

Frequency: Intra-rater reliability was examined in two studies with a total of 73 stroke patients [44,45]. They were aged between 40 and 66 years. The reliability ranged between ICC 0.87 and ICC 0.95, with a weighted mean average ICC of 0.92.

Stiffness: The intra-rater reliability was examined in a study with 30 healthy male subjects [62]. The subjects were aged between 73 and 81 years. The reliability was ICC 0.97. In addition, a total of 73 patients with stroke were examined in two studies [44,45]. These were aged between 40 and 66 years. The reliability ranged between ICC 0.88 and ICC 0.96 and came to a weighted mean average ICC of 0.93. In addition, eight male Parkinson’s patients were examined in one study [62]. This came to a reliability of 0.97.

Decrement: The intra-rater reliability was examined in two studies with a total of 73 stroke patients [44,45]. These were aged between 40 and 66 years. The reliability ranged between ICC 0.89 and ICC 0.93, with a weighted mean average ICC of 0.91.

#### 3.3.5. Brachioradialis Muscle

The diagnostic value of the brachioradialis muscle was evaluated in three studies [35,40,64]. The parameters frequency, stiffness, decrement, and creep were recorded.

Frequency: The intra-rater reliability was examined in a study with 17 healthy male subjects [64]. The subjects were aged between 20 and 22 years. The reliability was ICC 0.96. In addition, 28 patients with stroke were examined in a study [35]. These had an ICC value of 0.82.

The inter-rater reliability was examined in a study with 29 patients with stroke [40]. The subjects were aged between 30 and 83 years, with 5 females and 24 males. The reliability was ICC 0.86.

Stiffness: The intra-rater reliability was examined in a study with 17 healthy male subjects [64]. The subjects were aged between 20 and 22 years. The reliability was ICC 0.92.

The inter-rater reliability was examined in a study with 29 patients with stroke [40]. The subjects were aged between 30 and 83 years, and 5 were female and 24 were male. The reliability was ICC 0.90

Decrement: The intra-rater reliability was examined in a study with 17 healthy male subjects [64]. The subjects were aged between 20 and 22 years. The reliability was ICC 0.97.

The inter-rater reliability was examined in a study with 29 patients with stroke [40]. The subjects were aged between 30 and 83 years, and 5 were female and 24 were male. The reliability was ICC 0.94.

Creep: The inter-rater reliability was examined in a study with 29 patients with stroke [40]. The subjects were aged between 30 and 83 years, and 5 were female and 24 were male. The reliability was ICC 0.81.

#### 3.3.6. Flexor Carpi Ulnaris Muscle

The diagnostic value of the flexor carpi ulnaris muscle was examined in three studies [45,65,66]. The parameters evaluated were frequency, stiffness, and decrement. The inter-rater reliability was not examined in any of the studies.

Frequency: Intra-rater reliability was examined in a study with a total of 70 healthy test subjects [65]. The subjects were aged between 18 and 35 years. The reliability was ICC 0.82. In addition, a total of 128 patients with stroke were examined in two studies [45,66]. They were aged between 44 and 76 years, and 52 were female and 76 male. They achieved an ICC value of 0.89 to 0.93 and thus a weighted mean average ICC of 0.92.

Stiffness: Intra-rater reliability was examined in a study with a total of 70 healthy test subjects [65]. The subjects were aged between 18 and 35 years. The reliability was 0.90. In addition, a total of 128 patients with stroke were examined in two studies [45,66]. They were aged between 44 and 76 years, and 52 were female and 76 male. They had an ICC value of 0.87 to 0.92 and thus a weighted mean average ICC of 0.90.

Decrement: Intra-rater reliability was examined in a study with a total of 70 healthy test subjects [65]. The subjects were aged between 18 and 35 years. The reliability was ICC 0.86. In addition, a total of 128 patients with stroke were examined in two studies [45,66]. They were aged between 44 and 76 years, and 52 were female and 76 male. They achieved an ICC value of 0.89 to 0.90 and thus a weighted mean average ICC of 0.89.

#### 3.3.7. Flexor Carpi Radialis Muscle

Three studies [45,55,66] assessed the diagnostic value of flexor carpi radialis muscle. The parameters frequency, stiffness, and decrement were assessed. Inter-rater reliability was not examined in any study.

Frequency: The inter-rater reliability was examined in two studies with 128 patients with stroke [45,66]. The subjects were aged between 44 and 76 years, with 52 females and 76 males. The reliability ranged between ICC 0.93 and ICC 0.95 and came to a weighted mean average ICC of 0.95. In addition, 30 patients with Parkinson’s disease were examined in one study. They achieved an ICC value of 0.83 [55].

Stiffness: The inter-rater reliability was examined in two studies with 128 patients with stroke [45,66]. The subjects were aged between 44 and 76 years, with 52 females and 76 males. The reliability ranged between ICC 0.92 and ICC 0.94, with a weighted mean average ICC of 0.93. In addition, 30 patients with Parkinson’s disease were examined in one study. These patients achieved an ICC value of 0.86 [55].

Decrement: The inter-rater reliability was examined in two studies with 128 patients with stroke [45,66]. The subjects were aged between 44 and 76 years, with 52 females and 76 males. The reliability ranged between ICC 0.92 and ICC 0.93, with a weighted mean average ICC of 0.92. In addition, 30 patients with Parkinson’s disease were examined in one study. These patients achieved an ICC value of 0.85 [55].

#### 3.3.8. Extensor Carpi Radialis Brevis Muscle

One study [65] assessed the diagnostic value of extensor carpi radialis brevis muscle. The parameters frequency, stiffness, and decrement were assessed. Inter-rater reliability was not examined in any study.

Frequency: Intra-rater reliability was examined in a study with a total of 70 healthy test subjects [65]. The subjects were aged between 18 and 36 years. The reliability was ICC 0.98.

Stiffness: Intra-rater reliability was examined in a study with a total of 70 healthy test subjects [65]. The subjects were aged between 18 and 36 years. The reliability was ICC 0.92.

Decrement: The intra-rater reliability was examined in a study with a total of 70 healthy test subjects [65]. The subjects were aged between 18 and 36 years. The reliability was ICC 0.56.

#### 3.3.9. Extensor Digitorum Muscle

Two studies [45,66] assessed the diagnostic value of extensor digitorum muscle. The parameters frequency, stiffness, and decrement were assessed. Inter-rater reliability was not examined in any study.

Frequency: The inter-rater reliability was examined in two studies with 128 patients with stroke [45,66]. The subjects were aged between 44 and 76 years, with 52 females and 76 males. The reliability ranged between ICC 0.86 and ICC 0.93, with a weighted mean average ICC of 0.90.

Stiffness: The inter-rater reliability was examined in two studies with 128 patients with stroke [45,66]. The subjects were aged between 44 and 76 years, with 52 females and 76 males. The reliability ranged between ICC 0.86 and ICC 0.92, with a weighted mean average ICC of 0.90.

Decrement: The inter-rater reliability was examined in two studies with 128 patients with stroke [45,66]. The subjects were aged between 44 and 76 years, with 52 females and 76 males. The reliability ranged between ICC 0.75 and ICC 0.86, with a weighted mean average ICC of 0.81.

### 3.4. Muscles of Other Anatomical Regions

#### 3.4.1. Masseter Muscle

The diagnostic value of the masseter muscle was evaluated in three studies [67,68,69].

Frequency: Intra-rater reliability was examined in a study with a total of 16 healthy test subjects [67]. There were 10 females and 6 males. The subjects were aged between 18 and 20 years. The reliability was ICC 0.76.

Inter-rater reliability was examined in a study with a total of 16 healthy test subjects [67]. There were 10 females and 6 males. The subjects were aged between 18 and 20 years. The reliability was ICC 0.72.

Stiffness: Intra-rater reliability was examined in two studies with 36 healthy test subjects [67,68]. There were 20 females and 16 males. The reliability ICC ranged between 0.78 to 0.86 and came to a weighted mean average ICC of 0.82. In addition, 20 patients with stroke were examined in a study [69]. The patients were aged between 39 and 57 years, with 10 females and 10 males. They had an ICC value of 0.99.

The inter-rater reliability was examined in two studies with 36 healthy test subjects [67,68]. There were 20 females and 16 males. The reliability ICC ranged between 0.76 to 0.95 and came to a weighted mean average ICC of 0.87. In addition, 20 patients with stroke were examined in a study [69]. The patients were aged between 39 and 57 years, with 10 females and 10 males. They had an ICC value of 0.99.

Decrement: Intra-rater reliability was examined in a study with a total of 16 healthy test subjects [67]. There were 10 females and 6 males. The subjects were aged between 18 and 20 years. The reliability was ICC 0.88.

Inter-rater reliability was examined in a study with a total of 16 healthy test subjects [67]. There were 10 females and 6 males. The subjects were aged between 18 and 20 years. The reliability was ICC 0.82.

Relaxation: Intra-rater reliability was examined in a study with a total of 16 healthy test subjects [67]. There were 10 females and 6 males. The subjects were aged between 18 and 20 years. The reliability was ICC 0.66.

Inter-rater reliability was examined in a study with a total of 16 healthy test subjects [67]. There were 10 females and 6 males. The subjects were aged between 18 and 20 years. The reliability was ICC 0.76.

Creep: Intra-rater reliability was examined in a study with a total of 16 healthy test subjects [67]. There were 10 females and 6 males. The subjects were aged between 18 and 20 years. The reliability was ICC 0.71.

Inter-rater reliability was examined in a study with a total of 16 healthy test subjects [67]. There were 10 females and 6 males. The subjects were aged between 18 and 20 years. The reliability was ICC 0.82.

#### 3.4.2. Splenius Capitis Muscle

The diagnostic value of the splenius capitis muscle was evaluated in one study [47]. The parameters stiffness, frequency and decrement were recorded.

Frequency: Intra-rater reliability was investigated in a study with 20 healthy volunteers [47]. There were 10 females and 10 males. The reliability achieved an ICC of 0.75.

The inter-rater reliability was examined in a study with 20 healthy test subjects [47]. There were 10 females and 10 males. The reliability achieved an ICC of 0.80.

Stiffness: The intra-rater reliability was examined in a study with 20 healthy test subjects [47]. There were 10 females and 10 males. The reliability achieved an ICC of 0.57.

The inter-rater reliability was examined in a study with 20 healthy test subjects [47]. There were 10 females and 10 males. The reliability achieved an ICC of 0.73.

Decrement: The intra-rater reliability was examined in a study with 20 healthy test subjects [47]. There were 10 females and 10 males. The reliability achieved an ICC of 0.94.

The inter-rater reliability was examined in a study with 20 healthy test subjects [47]. There were 10 females and 10 males. The reliability achieved an ICC of 0.77.

#### 3.4.3. Sternocleidomastoideus Muscle

The diagnostic value of the sternocleidomastoid muscle was evaluated in two studies [67,70]. The parameters stiffness, frequency and decrement were recorded.

Frequency: The intra-rater reliability was examined in a study with a total of 16 healthy test subjects [67]. The reliability was ICC 0.74. In addition, a total of 22 post-mastectomy patients were examined in one study [70]. These achieved an ICC value of 0.87.

The inter-rater reliability was examined in a study with a total of 16 healthy test subjects [67]. The reliability ICC was 0.81. In addition, a total of 22 patients were examined post mastectomy in one study [70]. These achieved an ICC value of 0.65.

Stiffness: The intra-rater reliability was examined in a study with a total of 16 healthy test subjects [67]. The reliability was ICC 0.85. In addition, a total of 22 post-mastectomy patients were examined in one study [70]. These achieved an ICC value of 0.93.

The inter-rater reliability was examined in a study with a total of 16 healthy test subjects [67]. The reliability was 0.78. In addition, a total of 22 patients were examined post mastectomy in one study [70]. These achieved an ICC value of 0.87.

Decrement: The intra-rater reliability was examined in a study with a total of 16 healthy test subjects [67]. The reliability was ICC 0.86. In addition, a total of 22 post-mastectomy patients were examined in one study [70]. These achieved an ICC value of 0.94.

The inter-rater reliability was examined in a study with a total of 16 healthy test subjects [67]. The reliability was ICC 0.89. In addition, a total of 22 patients were examined in a study post mastectomy [70]. These achieved an ICC value of 0.92.

Relaxation: Intra-rater reliability was examined in a study with a total of 16 healthy test subjects [67]. There were 10 females and 6 males. The subjects were aged between 18 and 20 years. The reliability was ICC 0.74.

Inter-rater reliability was examined in a study with a total of 16 healthy test subjects [67]. There were 10 females and 6 males. The subjects were aged between 18 and 20 years. The reliability was ICC 0.65.

Creep: Intra-rater reliability was examined in a study with a total of 16 healthy test subjects [67]. There were 10 females and 6 males. The subjects were aged between 18 and 20 years. The reliability was ICC 0.52.

Inter-rater reliability was examined in a study with a total of 16 healthy test subjects [67]. There were 10 females and 6 males. The subjects were aged between 18 and 20 years. The reliability was ICC 0.73.

#### 3.4.4. Trapezius Muscle

The diagnostic value of the trapezius muscle was evaluated in three studies [67,71,72].

Frequency: Intra-rater reliability was examined in a study with a total of 16 healthy test subjects [67]. There were 10 females and 6 males. The subjects were aged between 18 and 20 years. The reliability was ICC 0.81.

Inter-rater reliability was examined in the same study with a total of 16 healthy test subjects. There were 10 females and 6 males. The subjects were aged between 18 and 20 years. The reliability was ICC 0.87.

Stiffness: Intra-rater reliability was examined in two studies with a total of 36 healthy test subjects [67,71]. The reliability ICC ranged between 0.82 and 0.97, with a weighted mean average ICC of 0.90. In addition, a total of 24 patients with neck and shoulder pain were examined in one study [72]. Only young men were examined. They achieved an ICC value of 0.75.

Inter-rater reliability was examined in two studies with a total of 36 healthy test subjects [67,71]. The reliability ranged between ICC 0.79 and ICC 0.97 and came to a weighted mean average ICC of 0.89.

Decrement: Intra-rater reliability was examined in a study with a total of 16 healthy test subjects [67]. There were 10 females and 6 males. The subjects were aged between 18 and 20 years. The reliability was ICC 0.76.

Inter-rater reliability was examined in the same study with a total of 16 healthy test subjects. There were 10 females and 6 males. The subjects were aged between 18 and 20 years. The reliability was ICC 0.93.

Relaxation: Intra-rater reliability was examined in a study with a total of 16 healthy test subjects [67]. There were 10 females and 6 males. The subjects were aged between 18 and 20 years. The reliability was ICC 0.74.

Inter-rater reliability was examined in the same study with a total of 16 healthy test subjects. There were 10 females and 6 males. The subjects were aged between 18 and 20 years. The reliability was ICC 0.65.

Creep: Intra-rater reliability was examined in a study with a total of 16 healthy test subjects [67]. There were 10 females and 6 males. The subjects were aged between 18 and 20 years. The reliability was ICC 0.52.

Inter-rater reliability was examined in the same study with a total of 16 healthy test subjects. There were 10 females and 6 males. The subjects were aged between 18 and 20 years. The reliability was ICC 0.50.

#### 3.4.5. Pectoralis Major Muscle

The diagnostic value of the pectoralis major muscle was evaluated in one study [70]. The stiffness parameter was measured. No healthy subjects were examined.

Stiffness: The intra-rater reliability was examined in a study with 22 patients post mastectomy [70]. The reliability was ICC 0.85.

Intra-rater reliability was investigated in a study with 22 patients post mastectomy [70]. The reliability was ICC 0.34.

#### 3.4.6. Cervical Extensor Muscle

One study [67] assessed the diagnostic value of cervical extensor muscle. The parameters frequency, stiffness, decrement, frequency, and creep were recorded. In addition, only healthy test subjects were examined. All subsequent results pertain to the aforementioned study.

Frequency: Intra-rater reliability was examined in a study with a total of 16 healthy test subjects. There were 10 females and 6 males. The subjects were aged between 18 and 20 years. The reliability was ICC 0.67.

Inter-rater reliability was examined in a study with a total of 16 healthy test subjects. There were 10 females and 6 males. The subjects were aged between 18 and 20 years. The reliability was ICC 0.93.

Stiffness: Intra-rater reliability was examined in a study with a total of 16 healthy test subjects. There were 10 females and 6 males. The subjects were aged between 18 and 20 years. The reliability was ICC 0.77.

Inter-rater reliability was examined in a study with a total of 16 healthy test subjects. There were 10 females and 6 males. The subjects were aged between 18 and 20 years. The reliability was ICC 0.95.

Decrement: Intra-rater reliability was examined in a study with a total of 16 healthy test subjects. There were 10 females and 6 males. The subjects were aged between 18 and 20 years. The reliability was ICC 0.82.

Inter-rater reliability was examined in a study with a total of 16 healthy test subjects. There were 10 females and 6 males. The subjects were aged between 18 and 20 years. The reliability was ICC 0.78.

Relaxation: Intra-rater reliability was examined in a study with a total of 16 healthy test subjects. There were 10 females and 6 males. The subjects were aged between 18 and 20 years. The reliability was ICC 0.85.

Inter-rater reliability was examined in a study with a total of 16 healthy test subjects. There were 10 females and 6 males. The subjects were aged between 18 and 20 years. The reliability was ICC 0.91.

Creep: Intra-rater reliability was examined in a study with a total of 16 healthy test subjects. There were 10 females and 6 males. The subjects were aged between 18 and 20 years. The reliability was ICC 0.79.

Inter-rater reliability was examined in a study with a total of 16 healthy test subjects. There were 10 females and 6 males. The subjects were aged between 18 and 20 years. The reliability was ICC 0.81.

#### 3.4.7. Erector Spinae Muscle

The diagnostic value of the erector spinae muscle was evaluated in three studies [48,73,74]. The parameters frequency, stiffness, and decrease were recorded.

Frequency: Intra-rater reliability was examined in a study with a total of 24 healthy test subjects [73]. The subjects were aged between 26 and 50 years, and 13 were female and 11 were male. The reliability was ICC 0.98.

The inter-rater reliability was examined in the same study with a total of 24 healthy test subjects. The subjects were aged between 26 and 50 years, and 13 were female and 11 were male. The reliability was ICC 0.94.

Stiffness: The intra-rater reliability was examined in two studies with a total of 54 healthy test subjects [48,73]. The reliability ranged between ICC 0.97 and ICC 0.99, with a weighted mean average ICC of 0.98. In addition, 20 patients with chronic back pain were examined in one study [74]. They were aged between 26 and 64 years. They achieved an ICC value of 0.90.

The inter-rater reliability was examined in two studies with a total of 54 healthy test subjects [48,73]. The reliability ranged between 0.94 and 0.99, with a mean value of 0.97. In addition, 20 patients with chronic back pain were examined in one study [74]. They were aged between 26 and 64 years. They achieved an ICC value of 0.99.

Decrement: Intra-rater reliability was examined in a study with a total of 24 healthy test subjects [73]. The subjects were aged between 26 and 50 years, and 13 were female and 11 were male. The reliability was 0.93.

The inter-rater reliability was examined in the same study with a total of 24 healthy test subjects. The subjects were aged between 26 and 50 years, with 13 females and 11 males. The reliability was 0.82.

#### 3.4.8. Lumbar Extensor Muscles

One study [75] assessed the diagnostic value of lumbar extensor muscle. The parameters frequency, stiffness, and decrement were assessed. The intra-rater reliability was not examined in any study. All subsequent results pertain to the aforementioned study.

Frequency: The inter-rater reliability was examined in a study with a total of 40 healthy test subjects. The subjects were aged between 50 and 80 years, with 20 females and 20 males. The reliability was ICC 0.93. In the same study, 40 patients with chronic back pain were also examined. These patients had an ICC value of 0.92.

Stiffness: The inter-rater reliability was examined in a study with a total of 40 healthy test subjects. The subjects were aged between 50 and 80 years, with 20 females and 20 males. The reliability was ICC 0.95. In the same study, 40 patients with chronic back pain were also examined. These patients had an ICC value of 0.94.

Decrement: The inter-rater reliability was examined in a study with a total of 40 healthy test subjects. The subjects were aged between 50 and 80 years, with 20 females and 20 males. The reliability was ICC 0.65. In the same study, 40 patients with chronic back pain were also examined. These patients had an ICC value of 0.90.

#### 3.4.9. Perineal Muscle

One study [76] evaluated the diagnostic value of the perineal muscle. The parameters frequency, decrement, creep, and relaxation were recorded. All subsequent results pertain to the aforementioned study.

Frequency: Intra-rater reliability was investigated in a study with 43 healthy female volunteers. The test subjects were aged between 15 and 50 years. The reliability was ICC 0.80. In addition, 32 patients with vulvodynia were examined in the same study. These patients had an ICC value of 0.95.

The inter-rater reliability was examined in a study with 43 healthy test subjects. The test subjects were aged between 15 and 50 years. The reliability was ICC 0.73. In addition, 32 patients with vulvodynia were examined in the same study. These patients had an ICC value of 0.94.

Decrement: The intra-rater reliability was examined in a study with 43 healthy test subjects. The test subjects were aged between 15 and 50 years. The reliability was ICC 0.87. In addition, 32 patients with vulvodynia were examined in the same study. These patients had an ICC value of 0.95.

The inter-rater reliability was examined in a study with 43 healthy test subjects. The test subjects were aged between 15 and 50 years. The reliability was ICC 0.85. In addition, 32 patients with vulvodynia were examined in the same study. These had an ICC value of 0.92.

Relaxation: Intra-rater reliability was investigated in a study with 43 healthy female volunteers. The test subjects were aged between 15 and 50 years. The reliability was 0.87. In addition, 32 patients with vulvodynia were examined in the same study. These patients had an ICC value of 0.96.

The inter-rater reliability was examined in a study with 43 healthy test subjects. The test subjects were aged between 15 and 50 years. The reliability was ICC 0.89. In addition, 32 patients with vulvodynia were examined in the same study. These had an ICC value of 0.92.

Creep: The intra-rater reliability was examined in a study with 43 healthy test subjects. The test subjects were aged between 15 and 50 years. The reliability was ICC 0.91. In addition, 32 patients with vulvodynia were examined in the same study. These patients had an ICC value of 0.88.

The inter-rater reliability was examined in a study with 43 healthy test subjects. The test subjects were aged between 15 and 50 years. The reliability was ICC 0.89. In addition, 32 patients with vulvodynia were examined in the same study. They achieved an ICC value of 0.93.

#### 3.4.10. Pelvic Floor Muscle

One study [77] investigated the diagnostic value of the pelvic floor muscle. The parameters frequency, stiffness, decrement, creep, and relaxation were recorded. All subsequent results pertain to the aforementioned study.

Frequency: The intra-rater reliability was examined in a study with 40 healthy female test subjects. The reliability was ICC 0.81. In addition, 38 patients with urinary incontinence were examined in the same study. They achieved an ICC value of 0.89.

The inter-rater reliability was examined in a study with 40 healthy female test subjects. The reliability was ICC 0.89. In addition, 38 patients with urinary incontinence were examined in the same study. They achieved an ICC value of 0.83.

Stiffness: The intra-rater reliability was examined in a study with 40 healthy female test subjects. The reliability was ICC 0.83. In addition, 38 patients with urinary incontinence were examined in the same study. They achieved an ICC value of 0.82.

The inter-rater reliability was examined in a study with 40 healthy female test subjects. The reliability was ICC 0.82. In addition, 38 patients with urinary incontinence were examined in the same study. They achieved an ICC value of 0.80.

Decrement: The intra-rater reliability was examined in a study with 40 healthy test subjects. The reliability was ICC 0.75. In addition, 38 patients with urinary incontinence were examined in the same study. They achieved an ICC value of 0.78.

The inter-rater reliability was examined in a study with 40 healthy female test subjects. The reliability was ICC 0.87. In addition, 38 patients with urinary incontinence were examined in the same study. They achieved an ICC value of 0.78.

Relaxation: The intra-rater reliability was examined in a study with 40 healthy test subjects. The reliability was ICC 0.70. In addition, 38 patients with urinary incontinence were examined in this study. They achieved an ICC value of 0.94.

The inter-rater reliability was examined in a study with 40 healthy female test subjects. The reliability was ICC 0.73. In addition, 38 patients with urinary incontinence were examined in this study. They achieved an ICC value of 0.78.

Creep: The intra-rater reliability was examined in a study with 40 healthy test subjects. The reliability was ICC 0.63. In addition, 38 patients with urinary incontinence were examined in this study. They achieved an ICC value of 0.85.

The inter-rater reliability was examined in a study with 40 healthy female test subjects. The reliability was ICC 0.40. In addition, 38 patients with urinary incontinence were examined in this study. They achieved an ICC value of 0.42.

## 4. Discussion

The results evince noteworthy values across the tripartite domains encompassing the lower limb, upper limb, and other anatomical regions. Subsequently, a discrete discussion shall ensue pertaining to individual muscles and ligaments.

Rectus Femoris Muscle: The rectus femoris muscle demonstrated good diagnostic potential, especially in terms of frequency and stiffness. The intra-rater reliability for frequency ranged from 0.63 to 0.99, with a mean of 0.87, and for stiffness, it ranged from 0.78 to 0.99, with a mean of 0.92. The inter-rater reliability was also promising, with mean values of 0.85 for frequency and 0.90 for stiffness. These findings suggest that rectus femoris muscle parameters can be consistently measured, making them reliable for diagnostic purposes.

Vastus Lateralis Muscle: The vastus lateralis muscle exhibited favorable diagnostic characteristics, particularly in terms of frequency and stiffness. The intra-rater reliability for frequency ranged from 0.65 to 0.82, with a mean of 0.735, and for stiffness, it ranged from 0.65 to 0.97, with a mean of 0.813. The inter-rater reliability was consistently high, with mean values of 0.95 for frequency and 0.938 for stiffness. Despite the lack of information on decrement, the vastus lateralis muscle appears to be a reliable diagnostic marker.

Vastus Medialis Muscle: The vastus medialis muscle was evaluated in fewer studies, but the available data suggest good diagnostic potential. The intra-rater reliability for frequency and stiffness ranged from 0.79 to 0.80 and 0.62 to 0.79, respectively, indicating consistent measurements. The inter-rater reliability was also high, with mean values of 0.94 for frequency and 0.94 for stiffness. Further studies are warranted to explore additional parameters and validate these findings.

Biceps Femoris Muscle: The biceps femoris muscle demonstrated excellent diagnostic reliability, especially in terms of frequency and stiffness. The intra-rater reliability for frequency and stiffness ranged from 0.99 to 0.92 and 0.86 to 0.99, respectively. The inter-rater reliability was also robust, with mean values of 0.81 for frequency and 0.76 for stiffness. The data on decrement, creep, and relaxation further support the muscle’s diagnostic value.

Patellar Ligament: The patellar ligament exhibited consistent diagnostic potential across studies. The intra-rater and inter-rater reliability for frequency, stiffness, and decrement were consistently high, with mean values ranging from 0.788 to 0.915. These findings suggest that the patellar ligament can be reliably assessed for diagnostic purposes in healthy individuals.

Gastrocnemius Medialis Muscle: The gastrocnemius medialis muscle showed promising diagnostic reliability. The intra-rater reliability for frequency, stiffness, decrement, creep, and relaxation ranged from 0.78 to 0.99, with mean values of 0.873, 0.906, 0.81, 0.885, and 0.89, respectively. The inter-rater reliability was similarly robust, with mean values ranging from 0.873 to 0.98. Despite limited data on relaxation and no information on inter-rater reliability for certain parameters, the gastrocnemius medialis muscle appears to be a valuable diagnostic marker.

Gastrocnemius Lateralis Muscle: The gastrocnemius lateralis muscle demonstrated good diagnostic reliability, especially in terms of stiffness. The intra-rater reliability for stiffness ranged from 0.84 to 0.99, with a mean of 0.923. Unfortunately, there is a lack of information on intra-rater reliability for other parameters and on inter-rater reliability. Further studies are needed to comprehensively assess the muscle’s diagnostic potential.

Soleus Muscle: The soleus muscle exhibited reliable diagnostic characteristics. The intra-rater reliability for frequency, stiffness, and decrement ranged from 0.90 to 0.93, 0.66 to 0.95, and 0.71 to 0.94, respectively. The inter-rater reliability was similarly robust, with mean values of 0.93, 0.943, and 0.817 for frequency, stiffness, and decrement, respectively. These findings suggest that the soleus muscle can be consistently assessed for diagnostic purposes in healthy individuals.

Tibialis Anterior Muscle: The tibialis anterior muscle demonstrated consistent diagnostic reliability across various parameters. The intra-rater reliability for frequency, stiffness, decrement, and creep ranged from 0.80 to 0.94, 0.85 to 0.98, 0.95, and 0.79, respectively. The inter-rater reliability was also robust, with mean values of 0.835, 0.86, 0.79, and 0.73 for frequency, stiffness, decrement, and creep, respectively. These findings highlight the tibialis anterior muscle’s potential as a reliable diagnostic marker.

Achilles Tendon: The Achilles tendon displayed reliable diagnostic characteristics. The intra-rater reliability for frequency, stiffness, decrement, creep, and relaxation ranged from 0.77 to 0.96, 0.74 to 0.96, 0.68 to 0.94, 0.89, and 0.82, respectively. The inter-rater reliability was consistently high, with mean values of 0.80, 0.92, 0.81, 0.92, and 0.92 for frequency, stiffness, decrement, creep, and relaxation, respectively. These findings suggest that the Achilles tendon can be consistently assessed for diagnostic purposes.

Plantar Fascia: The plantar fascia, although evaluated in a single study, demonstrated promising diagnostic reliability. The intra-rater reliability for frequency, stiffness, and decrement ranged from 0.77 to 0.96, 0.96, and 0.55, respectively. The inter-rater reliability was also robust, with values of 0.92, 0.97, and 0.90 for frequency, stiffness, and decrement, respectively. Further studies are needed to confirm these findings, but the initial results suggest the plantar fascia potential as a reliable diagnostic marker in healthy individuals.

Infraspinatus Muscle: The diagnostic value of the infraspinatus muscle was primarily evaluated based on frequency, stiffness, and decrement. The intra-rater reliability for frequency and decrement was only assessed in a study with patients experiencing shoulder pain. The results indicated excellent reliability with ICC values of 0.97 for frequency and 0.96 for decrement. The inter-rater reliability, assessed in the same study, demonstrated a high ICC value of 0.908 for frequency and 0.91 for decrement. However, the lack of intra-rater reliability assessment in healthy subjects limits the generalizability of these findings.

Deltoideus Muscle: The deltoideus muscle’s diagnostic value was assessed through parameters such as frequency, stiffness, and decrement. The intra-rater reliability for frequency, stiffness, and decrement showed high ICC values, ranging from 0.75 to 0.92. The inter-rater reliability was also substantial, with values ranging from 0.80 to 0.89. These findings, based on studies involving healthy subjects and stroke patients, suggest that the Deltoideus muscle parameters exhibit good reliability, making them potentially valuable for diagnostic purposes.

Biceps Brachii Muscle: The diagnostic value of the biceps brachii muscle was extensively evaluated in eleven studies, considering frequency, stiffness, decrement, and creep. The intra-rater reliability for frequency, stiffness, and decrement showed varying ICC values, with some studies demonstrating high reliability (e.g., ICC value of 0.93 for frequency in healthy subjects). The inter-rater reliability also displayed variability but generally fell within an acceptable range (mean value of 0.76 for frequency in healthy subjects). The inclusion of patients with stroke and paratonia introduced additional insights, indicating mixed reliability outcomes depending on the specific parameter and population.

Triceps Brachii Muscle: The diagnostic evaluation of the triceps brachii muscle involved studies focusing on frequency, stiffness, and decrement. The intra-rater reliability for frequency and decrement in stroke patients demonstrated high ICC values, ranging from 0.87 to 0.95. Stiffness exhibited an excellent reliability value of 0.97 in healthy male subjects and 0.965 in Parkinson’s patients. However, inter-rater reliability was not assessed in any study, limiting the comprehensive understanding of the muscle’s diagnostic potential.

Brachioradialis Muscle: The Brachioradialis muscle’s diagnostic value, evaluated in three studies, demonstrated high intra-rater reliability for frequency, stiffness, and decrement. The inter-rater reliability also indicated consistency, with ICC values ranging from 0.86 to 0.90. These findings suggest that the Brachioradialis muscle parameters could be reliable indicators for diagnostic purposes, especially considering the inclusion of both healthy subjects and stroke patients in the studies.

Flexor Carpi Ulnaris Muscle: The diagnostic assessment of the flexor carpi ulnaris muscle involved studies focusing on frequency, stiffness, and decrement. The intra-rater reliability was consistently high, with ICC values ranging from 0.82 to 0.90 in healthy subjects and stroke patients. These findings indicate that the flexor carpi ulnaris muscle parameters could provide reliable diagnostic information, although inter-rater reliability was not assessed in the available studies.

Flexor Carpi Radialis Muscle: The diagnostic value of the flexor carpi radialis muscle, based on three studies, demonstrated high intra-rater reliability for frequency, stiffness, and decrement. Inter-rater reliability, while not assessed, limits a comprehensive understanding of the muscle’s overall diagnostic potential.

Extensor Carpi Radialis Brevis Muscle: The diagnostic assessment of the extensor carpi radialis brevis muscle, based on one study, indicated high intra-rater reliability for frequency (0.98), stiffness (0.92), and decrement (0.56). These findings suggest that the muscle’s parameters could be reliable for diagnostic purposes, although further research is needed to confirm these results due to the limited available data.

Extensor Digitorum Muscle: The diagnostic evaluation of the extensor digitorum muscle, based on two studies, revealed high inter-rater reliability for frequency, stiffness, and decrement, with mean ICC values ranging from 0.813 to 0.903. These findings suggest that the muscle parameters could be reliable for diagnostic purposes, particularly in stroke patients.

Masseter Muscle: The masseter muscle exhibited good reliability in frequency and stiffness measurements, with ICC values ranging from 0.72 to 0.99. The decrement parameter also demonstrated high reliability, with ICC values of 0.82 and 0.88. However, relaxation and creep parameters displayed moderate reliability, indicating potential variability in these measurements. Overall, the masseter muscle shows promise as a reliable indicator for diagnostic purposes, particularly regarding frequency, stiffness, and decrement measurements. Additionally, the high reliability observed in patients with stroke underscores the muscle’s potential in clinical assessments beyond musculoskeletal evaluations.

Splenius Capitis Muscle: The diagnostic evaluation of the Splenius capitis muscle involved assessing stiffness, frequency, and decrement. While the reliability of frequency measurements ranged from 0.75 to 0.80, indicating moderate consistency, decrement exhibited a higher intra-rater reliability of 0.94. These findings suggest that decrement may be a more reliable parameter for diagnostic purposes in comparison to frequency.

Sternocleidomastoideus Muscle: the sternocleidomastoideus muscle demonstrated good reliability in frequency and stiffness measurements, with ICC values ranging from 0.74 to 0.93. However, the decrement parameter showed slightly lower reliability, especially in inter-rater assessments, with ICC values of 0.89 and 0.92. Additionally, relaxation and creep parameters exhibited moderate reliability. These results suggest that the sternocleidomastoideus muscle may be reliable for certain diagnostic measurements, but variability exists across different parameters. Notably, the inclusion of post-mastectomy patients in one study highlights the muscle’s relevance in clinical contexts, albeit with slightly lower reliability compared to healthy subjects.

Trapezius Muscle: The trapezius muscle also displayed good reliability in terms of frequency and stiffness, with intra- and inter-rater ICC values ranging from 0.79 to 0.97. However, the decrement parameter showed comparatively lower reliability, particularly in inter-rater assessments, with ICC values of 0.65 and 0.93. Additionally, relaxation and creep parameters exhibited moderate to low reliability. These findings suggest that while the trapezius muscle may be reliable for certain diagnostic measurements, such as frequency and stiffness, caution is warranted when considering other parameters.

Pectoralis Major Muscle: The pectoralis major muscle demonstrated varied reliability in stiffness measurements, with one study reporting a reliability of 0.85 and another with a lower reliability of 0.34. The inconsistency may be attributed to the specific patient population (post mastectomy) and warrants further investigation to establish its diagnostic utility.

Cervical Extensor Muscle: The cervical extensor muscle demonstrated moderate reliability across all parameters, with ICC values ranging from 0.67 to 0.95. While frequency and stiffness measurements showed relatively higher reliability, relaxation, and creep parameters exhibited lower reliability. These findings suggest that while the cervical extensor muscle may have diagnostic potential, variability in measurements should be considered when interpreting results.

Erector Spinae Muscle: The erector spinae muscle exhibited robust reliability across frequency, stiffness, and decrement measurements. Notably, patients with chronic back pain showed high ICC values, indicating that these parameters may have diagnostic significance in both healthy and clinical populations.

Lumbar Extensor Muscles: While the lumbar extensor muscles demonstrated high inter-rater reliability in frequency, stiffness, and decrement measurements, the absence of intra-rater reliability assessment limits the comprehensive understanding of its diagnostic potential. Further studies focusing on intra-rater reliability are needed to establish the consistency of measurements.

Perineal Muscle: The perineal muscle exhibited moderate to high reliability in frequency, decrement, creep, and relaxation measurements. The consistent reliability across multiple parameters suggests its potential as a diagnostic marker, especially in patients with vulvodynia.

Pelvic Floor Muscle: The pelvic floor muscle displayed varying reliability across parameters, with stiffness, and decrement measurements showing relatively higher reliability compared to frequency. These variations suggest the need for further investigation to determine the specific diagnostic value of each parameter.

Scar: One study [78] additionally evaluated both intra-rater and inter-rater reliability for scar assessment. The study involved 19 post-cesarean section patients aged between 21 and 40 years old. All five parameters were assessed, demonstrating ICC values ranging from 0.91 to 0.99, indicating high intra-rater and inter-rater reliability across all parameters. This implies that assessments of scar tissue can consistently provide reliable results, thus serving as valuable diagnostic indicators in this particular patient cohort.

Limitations: There are several limitations that warrant consideration. Firstly, there exists limited potential for generalizability, given that the majority of studies focus on healthy individuals, with fewer examinations conducted on patients with specific conditions such as stroke, paratonia, Parkinson’s disease, or post-mastectomy states. Consequently, the transferability of findings to diverse patient populations may be constrained. Furthermore, data pertaining to certain parameters such as creep, relaxation, and decrement are scant for some muscles, including the vastus medialis and infraspinatus, thus impeding a thorough understanding of their diagnostic potential. Additionally, methodological heterogeneity, encompassing sample sizes, population characteristics, and measurement protocols, may introduce inconsistencies in reliability assessments, thereby complicating direct inter-study comparisons. Some studies may also exhibit small sample sizes, potentially compromising the robustness and generalizability of their findings. The 95% confidence intervals from the primary studies were not extracted. The intra-rater reliability was assessed more frequently than inter-rater reliability; however, inter-rater reliability holds greater clinical relevance. Publication bias may be present, as studies with positive outcomes are typically favored for publication, potentially leading to an overestimation of the reliability of certain muscles and ligaments. Furthermore, longitudinal data are often lacking, with most studies providing cross-sectional analyses of reliability and failing to conduct longitudinal follow-ups to assess measurement stability over time. Addressing these limitations could augment the robustness and applicability of future research endeavors in this domain, furnishing clinicians with more dependable tools for diagnostic evaluation and treatment planning.

## 5. Conclusions

In conclusion, the intra-rater and inter-rater reliability across multiple parameters indicate that these structures can be consistently assessed in both healthy individuals and patients with specific conditions, such as spinal cord injuries, stroke, and Parkinson’s disease. These findings carry substantial implications for clinical practice, as the reliable measurement of muscle and ligament parameters can contribute to accurate diagnostic assessments, treatment planning, and monitoring of therapeutic interventions.

## Figures and Tables

**Table 1:** Presentation of the PICOS statement.

**Population**	Patients with musculoskeletal disorders, muscle problems, or healthy participants.
**Intervention**	Use of the MyotonPro measurement device for muscle assessment.
**Comparison**	Comparison with other/no assessment methods or devices.
**Outcome**	To assess the reliability of the MyotonPro.
**Study types**	Diagnostic cross-sectional studies, diagnostic case–control studies, inter-rater and intra-rater reliability studies, and randomized control trials.
**Language**	English, German

**Table 2:** Presentation of the distribution of participants across health conditions.

Health/Medical Condition	Number of Groups	Number of Study Participants
Healthy	33	1060
Stroke	6	217
Cerebral palsy	1	15
Vulvodynia	1	32
Parkinson’s disease	2	38
Paratonia	2	86
Spinal cord injuries	4	87
Shoulder pain	2	59
Post mastectomy	1	22
Post-cesarean section	1	19
Urinary incontinence	1	38
Overall	54	1673

## Data Availability

Additional data are available after a request from the corresponding authors.

## Supplementary Materials

The following supporting information can be downloaded at: https://www.mdpi.com/article/10.3390/medicina60060851/s1, Table S1: Inter-rater reliability; Table S2: Intra-rater reliability.

## Abbreviations

ICC	Intraclass Correlation Coefficients
PICOS	Population, Intervention, Comparison, Outcome, Study design
CI 95%	Confidence Interval
N/A	Not applicable
PRSIMA	Preferred Reporting Items for Systematic Reviews and Meta-Analyses

## References

[1] Gleim G.W., McHugh M.P. (1997). Flexibility and its effects on sports injury and performance. Sports Med..

[2] Brazier J., Maloney S., Bishop C., Read P.J., Turner A.N. (2019). Lower Extremity Stiffness: Considerations for Testing, Performance Enhancement, and Injury Risk. J. Strength. Cond. Res..

[3] Raiteri B.J., Cresswell A.G., Lichtwark G.A. (2018). Muscle-tendon length and force affect human tibialis anterior central aponeurosis stiffness in vivo. Proc. Natl. Acad. Sci. USA.

[4] McMahon T.A., Cheng G.C. (1990). The mechanics of running: How does stiffness couple with speed?. J. Biomech..

[5] Hobara H., Muraoka T., Omuro K., Gomi K., Sakamoto M., Inoue K., Kanosue K. (2009). Knee stiffness is a major determinant of leg stiffness during maximal hopping. J. Biomech..

[6] Kekelekis A., Nikolaidis P.T., Moore I.S., Rosemann T., Knechtle B. (2020). Risk Factors for Upper Limb Injury in Tennis Players: A Systematic Review. Int. J. Environ. Res. Public Health.

[7] Padua D.A., Arnold B.L., Perrin D.H., Gansneder B.M., Carcia C.R., Granata K.P. (2006). Fatigue, vertical leg stiffness, and stiffness control strategies in males and females. J. Athl. Train..

[8] Dugailly P.M., Coucke A., Salem W., Feipel V. (2018). Assessment of cervical stiffness in axial rotation among chronic neck pain patients: A trial in the framework of a non-manipulative osteopathic management. Clin. Biomech..

[9] Leppilahti J., Orava S. (1998). Total Achilles tendon rupture. A review. Sports Med..

[10] Flanagan E.P., Galvin L., Harrison A.J. (2008). Force production and reactive strength capabilities after anterior cruciate ligament reconstruction. J. Athl. Train..

[11] Pruyn E.C., Watsford M.L., Murphy A.J., Pine M.J., Spurrs R.W., Cameron M.L., Johnston R.J. (2012). Relationship between leg stiffness and lower body injuries in professional Australian football. J. Sports Sci..

[12] McHugh M.P., Connolly D.A., Eston R.G., Kremenic I.J., Nicholas S.J., Gleim G.W. (1999). The role of passive muscle stiffness in symptoms of exercise-induced muscle damage. Am. J. Sports Med..

[13] Lee W.C., Ng G.Y., Zhang Z.J., Malliaras P., Masci L., Fu S.N. (2020). Changes on Tendon Stiffness and Clinical Outcomes in Athletes Are Associated with Patellar Tendinopathy After Eccentric Exercise. Clin. J. Sport Med..

[14] Hess G.W. (2010). Achilles tendon rupture: A review of etiology, population, anatomy, risk factors, and injury prevention. Foot Ankle Spec..

[15] Maloney S.J., Richards J., Nixon D.G., Harvey L.J., Fletcher I.M. (2017). Do stiffness and asymmetries predict change of direction performance?. J. Sports Sci..

[16] Hamill J., Gruber A.H., Derrick T.R. (2014). Lower extremity joint stiffness characteristics during running with different footfall patterns. Eur. J. Sport Sci..

[17] Maquirriain J. (2012). Leg stiffness changes in athletes with Achilles tendinopathy. Int. J. Sports Med..

[18] Gracies J.M. (2005). Pathophysiology of spastic paresis. I: Paresis and soft tissue changes. Muscle Nerve.

[19] Thibaut A., Chatelle C., Ziegler E., Bruno M.A., Laureys S., Gosseries O. (2013). Spasticity after stroke: Physiology, assessment and treatment. Brain. Inj..

[20] Wu Z., Ye X., Ye Z., Hong K., Chen Z., Wang Y., Li C., Li J., Huang J., Zhu Y. (2022). Asymmetric Biomechanical Properties of the Paravertebral Muscle in Elderly Patients with Unilateral Chronic Low Back Pain: A Preliminary Study. Front. Bioeng. Biotechnol..

[21] Watsford M.L., Murphy A.J., McLachlan K.A., Bryant A.L., Cameron M.L., Crossley K.M., Makdissi M. (2010). A prospective study of the relationship between lower body stiffness and hamstring injury in professional Australian rules footballers. Am. J. Sports Med..

[22] Morgan G.E., Martin R., Williams L., Pearce O., Morris K. (2018). Objective assessment of stiffness in Achilles tendinopathy: A novel approach using the MyotonPRO. BMJ Open. Sport. Exerc. Med..

[23] Guduru R.K.R., Domeika A., Domeikienė A. (2022). Effect of Rounded and Hunched Shoulder Postures on Myotonometric Measurements of Upper Body Muscles in Sedentary Workers. Appl. Sci..

[24] Królikowska A., Reichert P., Karlsson J., Mouton C., Becker R., Prill R. (2023). Improving the reliability of measurements in orthopaedics and sports medicine. Knee Surg. Sports Traumatol. Arthrosc..

[25] Page M.J., Moher D., Bossuyt P.M., Boutron I., Hoffmann T.C., Mulrow C.D., Shamseer L., Tetzlaff J.M., Akl E.A., Brennan S.E. (2021). PRISMA 2020 explanation and elaboration: Updated guidance and exemplars for reporting systematic reviews. BMJ.

[26] Ardern C.L., Büttner F., Andrade R., Weir A., Ashe M.C., Holden S., Impellizzeri F.M., Delahunt E., Dijkstra H.P., Mathieson S. (2022). Implementing the 27 PRISMA 2020 Statement items for systematic reviews in the sport and exercise medicine, musculoskeletal rehabilitation and sports science fields: The PERSiST (implementing Prisma in Exercise, Rehabilitation, Sport medicine and SporTs science) guidance. Br. J. Sports Med..

[27] Prill R., Karlsson J., Ayeni O.R., Becker R. (2021). Author guidelines for conducting systematic reviews and meta-analyses. Knee Surg Sports Traumatol Arthrosc.

[28] Sterne J.A.C., Savović J., Page M.J., Elbers R.G., Blencowe N.S., Boutron I., Cates C.J., Cheng H.Y., Corbett M.S., Eldridge S.M. (2019). RoB 2, A revised tool for assessing risk of bias in randomised trials. BMJ.

[29] Campbell J.M., Klugar M., Ding S., Carmody D.P., Hakonsen S.J., Jadotte Y.T., White S., Munn Z., Aromataris E., Munn Z. (2020). Chapter 9, Diagnostic test accuracy systematic reviews. JBI Manual for Evidence Synthesis.

[30] Koo T.K., Li M.Y. (2016). A Guideline of Selecting and Reporting Intraclass Correlation Coefficients for Reliability Research. J. Chiropr. Med..

[31] Lidström A., Ahlsten G., Hirchfeld H., Norrlin S. (2009). Intrarater and interrater reliability of Myotonometer measurements of muscle tone in children. J. Child. Neurol..

[32] Mullix J., Warner M., Stokes M. (2012). Testing muscle tone and mechanical properties of rectus femoris and biceps femoris using a novel hand-held MyotonPRO device: Relative ratios and reliability. Working Papers in the Health Sciences.

[33] Aird L., Samuel D., Stokes M. (2012). Quadriceps muscle tone, elasticity and stiffness in older males: Reliability and symmetry using the MyotonPRO. Arch. Gerontol. Geriatr..

[34] Chen G., Wu J., Chen G., Lu Y., Ren W., Xu W., Xu X., Wu Z., Guan Y., Zheng Y. (2019). Reliability of a portable device for quantifying tone and stiffness of quadriceps femoris and patellar tendon at different knee flexion angles. PLoS ONE.

[35] Lo W.L.A., Zhao J.L., Chen L., Lei D., Huang D.F., Tong K.F. (2017). Between-days intra-rater reliability with a hand held myotonometer to quantify muscle tone in the acute stroke population. Sci. Rep..

[36] Ko C.Y., Choi H.J., Ryu J., Kim G. (2018). Between-day reliability of MyotonPRO for the non-invasive measurement of muscle material properties in the lower extremities of patients with a chronic spinal cord injury. J. Biomech..

[37] Fröhlich-Zwahlen A.K., Casartelli N.C., Item-Glatthorn J.F., Maffiuletti N.A. (2014). Validity of resting myotonometric assessment of lower extremity muscles in chronic stroke patients with limited hypertonia: A preliminary study. J. Electromyogr. Kinesiol..

[38] Agyapong-Badu S., Aird L., Bailey L., Mooney K., Mullix J., Warner M., Samuel D., Stokes M. (2013). Interrater reliability of muscle tone, stiffness and elasticity measurements of rectus femoris and biceps brachii in healthy young and older males. Working Papers in the Health Sciences.

[39] Bravo-Sánchez A., Abián P., Sánchez-Infante J., Ramírez-delaCruz M., Esteban-García P., Jiménez F., Abián-Vicén J. (2022). Five-Compressions Protocol as a Valid Myotonometric Method to Assess the Stiffness of the Lower Limbs: A Brief Report. Int. J. Environ. Res. Public Health.

[40] Lo W.L.A., Zhao J.L., Li L., Mao Y.R., Huang D.F. (2017). Relative and Absolute Interrater Reliabilities of a Hand-Held Myotonometer to Quantify Mechanical Muscle Properties in Patients with Acute Stroke in an Inpatient Ward. BioMed Res. Int..

[41] Lee Y., Kim M., Lee H. (2021). The Measurement of Stiffness for Major Muscles with Shear Wave Elastography and Myoton: A Quantitative Analysis Study. Diagnostics.

[42] Bravo-Sánchez A., Abián P., Sánchez-Infante J., Esteban-Gacía P., Jiménez F., Abián-Vicén J. (2021). Objective Assessment of Regional Stiffness in Vastus Lateralis with Different Measurement Methods: A Reliability Study. Sensors.

[43] Leonard C.T., Deshner W.P., Romo J.W., Suoja E.S., Fehrer S.C., Mikhailenok E.L. (2003). Myotonometer intra- and interrater reliabilities. Arch. Phys. Med. Rehabil..

[44] Chuang L.L., Wu C.Y., Lin K.C., Lur S.Y. (2012). Quantitative mechanical properties of the relaxed biceps and triceps brachii muscles in patients with subacute stroke: A reliability study of the myoton-3 myometer. Stroke Res. Treat..

[45] Chuang L.L., Lin K.C., Wu C.Y., Chang C.W., Chen H.C., Yin H.P., Wang L. (2013). Relative and absolute reliabilities of the myotonometric measurements of hemiparetic arms in patients with stroke. Arch. Phys. Med. Rehabil..

[46] Sohirad S., Wilson D., Waugh C., Finnamore E., Scott A. (2017). Feasibility of using a hand-held device to characterize tendon tissue biomechanics. PLoS ONE.

[47] Muckelt P.E., Warner M.B., Cheliotis-James T., Muckelt R., Hastermann M., Schoenrock B., Martin D., MacGregor R., Blottner D., Stokes M. (2022). Protocol and reference values for minimal detectable change of MyotonPRO and ultrasound imaging measurements of muscle and subcutaneous tissue. Sci. Rep..

[48] Kelly J.P., Koppenhaver S.L., Michener L.A., Proulx L., Bisagni F., Cleland J.A. (2018). Characterization of tissue stiffness of the infraspinatus, erector spinae, and gastrocnemius muscle using ultrasound shear wave elastography and superficial mechanical deformation. J. Electromyogr. Kinesiol..

[49] Li Y.P., Feng Y.N., Liu C.L., Zhang Z.J. (2020). Paraffin therapy induces a decrease in the passive stiffness of gastrocnemius muscle belly and Achilles tendon: A randomized controlled trial. Medicine.

[50] Feng Y.N., Li Y.P., Liu C.L., Zhang Z.J. (2018). Assessing the elastic properties of skeletal muscle and tendon using shearwave ultrasound elastography and MyotonPRO. Sci. Rep..

[51] Ge J.S., Chang T.T., Zhang Z.J. (2020). Reliability of Myotonometric Measurement of Stiffness in Patients with Spinal Cord Injury. Med. Sci. Monit..

[52] Taş S., Salkın Y. (2019). An investigation of the sex-related differences in the stiffness of the Achilles tendon and gastrocnemius muscle: Inter-observer reliability and inter-day repeatability and the effect of ankle joint motion. Foot.

[53] Albin S.R., Koppenhaver S.L., Bailey B., Blommel H., Fenter B., Lowrimore C., Smith A.C., McPoil T.G. (2019). The effect of manual therapy on gastrocnemius muscle stiffness in healthy individuals. Foot.

[54] Jiménez-Sánchez C., Ortiz-Lucas M., Bravo-Esteban E., Mayoral-Del Moral O., Herrero-Gállego P., Gómez-Soriano J. (2018). Myotonometry as a measure to detect myofascial trigger points: An inter-rater reliability study. Physiol. Meas..

[55] Agoriwo M.W., Muckelt P.E., Yeboah C.O., Sankah B.E.A., Agyapong-Badu S., Akpalu A., Stokes M. (2022). Feasibility and reliability of measuring muscle stiffness in Parkinson’s Disease using MyotonPRO device in a clinical setting in Ghana. Ghana Med. J..

[56] Schneebeli A., Falla D., Clijsen R., Barbero M. (2020). Myotonometry for the evaluation of Achilles tendon mechanical properties: A reliability and construct validity study. BMJ Open. Sport Exerc. Med..

[57] Chang T.T., Feng Y.N., Zhu Y., Liu C.L., Wang X.Q., Zhang Z.J. (2020). Objective Assessment of Regional Stiffness in Achilles Tendon in Different Ankle Joint Positions Using the MyotonPRO. Med. Sci. Monit..

[58] Liu C.L., Li Y.P., Wang X.Q., Zhang Z.J. (2018). Quantifying the Stiffness of Achilles Tendon: Intra- and Inter-Operator Reliability and the Effect of Ankle Joint Motion. Med. Sci. Monit..

[59] Roch M., Morin M., Gaudreault N. (2020). The MyotonPRO: A reliable tool for quantifying the viscoelastic properties of a trigger point on the infraspinatus in non-traumatic chronic shoulder pain. J. Bodyw. Mov. Ther..

[60] Van Deun B., Hobbelen J.S.M., Cagnie B., Van Eetvelde B., Van Den Noortgate N., Cambier D. (2018). Reproducible Measurements of Muscle Characteristics Using the MyotonPRO Device: Comparison Between Individuals with and without Paratonia. J. Geriatr. Phys. Ther..

[61] Drenth H., Zuidema S.U., Krijnen W.P., Bautmans I., van der Schans C., Hobbelen H. (2018). Psychometric Properties of the MyotonPRO in Dementia Patients with Paratonia. Gerontology.

[62] Marusiak J., Jarocka E., Jaskólska A., Jaskólski A. (2018). Influence of number of records on reliability of myotonometric measurements of muscle stiffness at rest and contraction. Acta. Bioeng. Biomech..

[63] Li Y.P., Liu C.L., Zhang Z.J. (2022). Feasibility of Using a Portable MyotonPRO Device to Quantify the Elastic Properties of Skeletal Muscle. Med. Sci. Monit..

[64] Jarocka E., Marusiak J., Kumorek M., Jaskólska A., Jaskólski A. (2012). Muscle stiffness at different force levels measured with two myotonometric devices. Physiol. Meas..

[65] Çevik Saldıran T., Kara İ., Kutlutürk Yıkılmaz S. (2022). Quantification of the forearm muscles mechanical properties using Myotonometer: Intra- and Inter-Examiner reliability and its relation with hand grip strength. J. Electromyogr. Kinesiol..

[66] Chuang L.L., Wu C.Y., Lin K.C. (2012). Reliability, validity, and responsiveness of myotonometric measurement of muscle tone, elasticity, and stiffness in patients with stroke. Arch. Phys. Med. Rehabil..

[67] Taş S., Yaşar Ü., Kaynak B.A. (2020). Interrater and Intrarater Reliability of a Handheld Myotonometer in Measuring Mechanical Properties of the Neck and Orofacial Muscles. J. Manip. Physiol. Ther..

[68] Yu J.F., Chang T.T., Zhang Z.J. (2020). The Reliability of MyotonPRO in Assessing Masseter Muscle Stiffness and the Effect of Muscle Contraction. Med. Sci. Monit..

[69] Song C., Yu Y.F., Ding W.L., Yu J.Y., Song L., Feng Y.N., Zhang Z.J. (2021). Quantification of the Masseter Muscle Hardness of Stroke Patients Using the MyotonPRO Apparatus: Intra- and Inter-Rater Reliability and Its Correlation with Masticatory Performance. Med. Sci. Monit..

[70] Yeo S.M., Kang H., An S., Cheong I., Kim Y., Hwang J.H. (2020). Mechanical Properties of Muscles around the Shoulder in Breast Cancer Patients: Intra-rater and Inter-rater Reliability of the MyotonPRO. PM&R.

[71] Liu C.L., Feng Y.N., Zhang H.Q., Li Y.P., Zhu Y., Zhang Z.J. (2018). Assessing the viscoelastic properties of upper trapezius muscle: Intra- and inter-tester reliability and the effect of shoulder elevation. J. Electromyogr. Kinesiol..

[72] Kisilewicz A., Janusiak M., Szafraniec R., Smoter M., Ciszek B., Madeleine P., Fernández-de-Las-Peñas C., Kawczyński A. (2018). Changes in Muscle Stiffness of the Trapezius Muscle After Application of Ischemic Compression into Myofascial Trigger Points in Professional Basketball Players. J. Hum. Kinet..

[73] Lohr C., Braumann K.M., Reer R., Schroeder J., Schmidt T. (2018). Reliability of tensiomyography and myotonometry in detecting mechanical and contractile characteristics of the lumbar erector spinae in healthy volunteers. Eur. J. Appl. Physiol..

[74] Li Y., Yu J., Zhang J., Zhang Z., Wang X. (2022). Quantifying the stiffness of lumbar erector spinae during different positions among participants with chronic low back pain. PLoS ONE.

[75] Wu Z., Zhu Y., Xu W., Liang J., Guan Y., Xu X. (2020). Analysis of Biomechanical Properties of the Lumbar Extensor Myofascia in Elderly Patients with Chronic Low Back Pain and That in Healthy People. BioMed Res. Int..

[76] Davidson M.J., Bryant A.L., Bower W.F., Frawley H.C. (2017). Myotonometry Reliably Measures Muscle Stiffness in the Thenar and Perineal Muscles. Physiother. Can..

[77] Rodrigues-de-Souza D.P., Alcaraz-Clariana S., García-Luque L., Carmona-Pérez C., Garrido-Castro J.L., Cruz-Medel I., Camargo P.R., Alburquerque-Sendín F. (2021). Absolute and Relative Reliability of the Assessment of the Muscle Mechanical Properties of Pelvic Floor Muscles in Women with and without Urinary Incontinence. Diagnostics.

[78] Gilbert I., Gaudreault N., Gaboury I. (2021). Intra- and inter-evaluator reliability of the MyotonPRO for the assessment of the viscoelastic properties of caesarean section scar and unscarred skin. Skin Res. Technol..

