# Supplementary material for: Evaluating the Reliability of MyotonPro in Assessing Muscle Properties: A Systematic Review of Diagnostic Test Accuracy
Source: Medicina (Kaunas). 2024 May 23;60(6):851. doi: 10.3390/medicina60060851 (PMC11205912; doi:10.3390/medicina60060851)
Supplement: Supplementary file 1 [file medicina-60-00851-s001.zip › medicina-3005957-supplementary.pdf]

Lettner et al., Evaluating the Reliability of MyotonPro in Assessing Muscle Properties: a Systematic Review of Diagnostic Test Accuracy

Supplementary File: Extra Web Material

1. Inter-rater Reliability……………………………………………………………………………………………… page 2

2. Intra-rater Reliability ……………………………………………………………………………………………… page 7

1. Inter-rater Reliability

Muscle	Author	N		Alter	Frequency	Stiffness	Decrement	Relaxation	Creep
M. rectus femoris	8	262			262	232	202	n/a	29
	Lidström et al. (2009)	30	Healthy and Cerebral palsy	11	ICC 0.85	n/a	n/a	n/a	n/a
	Mulix et al. (2012)	21	Healthy	20-35	ICC 0.81	ICC 0.83	ICC 0.87	n/a	n/a
	Aird et al. (2012)	20	Healthy	65-82	ICC 0.77	ICC 0.82	ICC 0.79	n/a	n/a
	Fröhlich-Zwahlen et al. (2014)	39	Healthy and Stroke	43-63	ICC 0.63	ICC 0.87	ICC 0.77	n/a	n/a
	Ambrse Lo et al. (2017)	29	Stroke	30-83	ICC 0.92	ICC 0.94	ICC 0.81	n/a	ICC 0.92
	Chen et al. (2019)	30	Healthy	23-27	ICC 0.92	ICC 0.96	n/a	n/a	n/a
	Badu et al. (2020)	42	Healthy	20-72	ICC 0.94	ICC 0.93	ICC 0.91	n/a	n/a
	Bravo-Sanchez et al. (2022)	52	Healthy	21-26	ICC 0.97	ICC 0.97	ICC 0.78	n/a	n/a
Weighted mean average					ICC 0.87	ICC 0.92	ICC 0.82	n/a	ICC 0.92
M. vastus lat.	4	175			122	175	91	n/a	n/a
	Fröhlich-Zwahlen et al. (2014)	39	Healthy and Stroke	43-63	ICC 0.91	ICC 0.92	ICC 0.83	n/a	n/a
	Chen et al. (2019)	30	Healthy	23-27	ICC 0.95	ICC 0.94	n/a	n/a	n/a
	Bravo-Sanchez et al. (2021)	53	Healthy	19-37	n/a	ICC 0.93	n/a	n/a	n/a
	Bravo-Sanchez et al. (2022)	52	Healthy	21-28	ICC 0.98	ICC 0.96	ICC 0.91	n/a	n/a
Weighted mean average					ICC 0.94	ICC 0.93	ICC 0.88	n/a	n/a
M. vastus med.	1	30			30	30	n/a	n/a	n/a
	Chen et al. (2019)	30	Healthy	23-27	ICC 0.94	ICC 0.95	n/a	n/a	n/a
Weighted mean average					ICC 0.94	ICC 0.95	n/a	n/a	n/a
M. biceps femoris	2	60			60	60	60	n/a	n/a
	Mulix et al. (2012)	21	Healthy	20-35	ICC 0.86	ICC 0.72	ICC 0.77	n/a	n/a
	Fröhlich-Zwahlen et al. (2014)	39	Healthy and Stroke	43-63	ICC 0.75	ICC 0.80	ICC 0.78	n/a	n/a

Weighted mean average					ICC 0.79	ICC 0.77	ICC 0.78	n/a	n/a
patellar ligament	3	102			102	102	72	n/a	n/a
	Chen et al. (2019)	30	Healthy	23-27	ICC 0.92	ICC 0.93	n/a	n/a	n/a
	Muckelt et al. (2022)	20	Healthy	26-32	ICC 0.52	ICC 0.86	ICC 0.63	n/a	n/a
	Bravo-Sanchez et al. (2022)	52	Healthy	21-28	ICC 0.95	ICC 0.95	ICC 0.95	n/a	n/a
Weighted mean average					ICC 0.86	ICC 0.93	ICC 0.86	n/a	n/a
M. gastrocnemius med.	4	92			39	92	39	n/a	n/a
	Fröhlich-Zwahlen et al. (2014)	39	Healthy and Stroke	43-63	ICC 0.69	ICC 0.77	ICC 0.62	n/a	n/a
	Li et al. (2018)	20	Healthy	n/a	n/a	ICC 0.86	n/a	n/a	n/a
	Tas et al. (2019)	19	Healthy	n/a	n/a	ICC 0.93	n/a	n/a	n/a
	Ge et al. (2021)	14	Spinal cord injuries	n/a	n/a	ICC 0.98	n/a	n/a	n/a
Weighted mean average					ICC 0.69	ICC 0.86	ICC 0.62	n/a	n/a
M. gastrocnemius lat.	4	153			n/a	153	n/a	n/a	n/a
	Leonard et al. (2003)	35	Healthy	22-42	n/a	ICC 0.86	n/a	n/a	n/a
	Li et al. (2018)	20	Healthy	n/a	n/a	ICC 0.92	n/a	n/a	n/a
	Albin et al. (2019)	84	Healthy	21-33	n/a	ICC 0.97	n/a	n/a	n/a
	Ge et al. (2021)	14	Spinal cord injuries	n/a	n/a	ICC 0.98	n/a	n/a	n/a
Weighted mean average					n/a	ICC 0.94	n/a	n/a	n/a
M. soleus	2	70			70	70	70	n/a	n/a
	Jimenez-Sanchez et al. (2018)	50	Healthy	17-33	ICC 0.92	ICC 0.94	ICC 0.90	n/a	n/a
	Muckelt et al. (2022)	20	Healthy	26-32	ICC 0.95	ICC 0.95	ICC 0.65	n/a	n/a
Weighted mean average					ICC 0.93	ICC 0.94	ICC 0.83	n/a	n/a
M. tibialis ant.	3	88			88	88	88	n/a	29
	Fröhlich-Zwahlen et al. (2014)	39	Healthy and Stroke	43-63	ICC 0.76	ICC 0.79	ICC 0.71	n/a	n/a
	Lo et al. (2017)	29	Stroke	30-83	ICC 0.89	ICC 0.87	ICC 0.74	n/a	ICC 0.90

	Muckelt et al. (2022)	20	Healthy	26-32	ICC 0.91	ICC 0.93	ICC 0.87	n/a	
Weighted mean average					ICC 0.84	ICC 0.85	ICC 0.76	n/a	ICC 0.90
Achilles tendon	7	163			20	163	20	n/a	n/a
	Liu et al. (2018)	20	Healthy	22-28	n/a	ICC 0.92	n/a	n/a	n/a
	Li et al. (2018)	20	Healthy	n/a	n/a	ICC 0.79	n/a	n/a	n/a
	Tas et al. (2019)	19	Healthy	n/a	n/a	ICC 0.89	n/a	n/a	n/a
	Schneebeli et al. (2020)	40	Healthy	n/a	n/a	ICC 0.86	n/a	n/a	n/a
	Ge et al. (2021)	14	Spinal cord injuries	n/a	n/a	ICC 0.98	n/a	n/a	n/a
	Chang et al. (2020)	30	Healthy	n/a	n/a	ICC 0.94	n/a	n/a	n/a
	Muckelt et al. (2022)	20	Healthy	26-32	ICC 0.52	ICC 0.86	ICC 0.77	n/a	n/a
Weighted mean average					ICC 0.52	ICC 0.89	ICC 0.77	n/a	n/a
Plantar fascia	1	20			20	20	20	n/a	n/a
	Muckelt et al. (2022)	20	Healthy	26-32	ICC 0.92	ICC 0.97	ICC 0.90	n/a	n/a
Weighted mean average					ICC 0.92	ICC 0.97	ICC 0.90	n/a	n/a

Muscle	Author	N		Alter	Frequency	Stiffness	Decrement	Relaxation	Creep
M. infraspinatu s	1	35			35	35	35	n/a	n/a
	Roch et al. (2020)	35	Shoulder pain	42	ICC 0.97	ICC 0.94	ICC 0.95	n/a	n/a
Weighted mean average					ICC 0.97	ICC 0.94	ICC 0.95	n/a	n/a
M. deltoideus	1	20			n/a	n/a	n/a	n/a	n/a
	Muckelt et al. (2022)	20	Healthy	26-32	ICC 0.80	ICC 0.89	ICC 0.77	n/a	n/a
Weighted mean average					ICC 0.80	ICC 0.89	ICC 0.77	n/a	n/a
M. biceps brachii	6	241			195	241	195	n/a	99
	Leonard et al. (2003)	35	Healthy	22-42	n/a	ICC 0.86	n/a	n/a	n/a
	Lo et al.(2017)	29	Stroke	30-83	ICC 0.77	ICC 0.71	ICC 0.82	n/a	ICC 0.76
	Drenth et al. (2017)	70	Paratonia		ICC 0.70	ICC 0.75	ICC 0.64	n/a	ICC 0.57
	Van Deun et al. (2018)	54	Healthy and Paratonia	24-98	ICC 0.61	ICC 0.62	ICC 0.63	n/a	n/a
	Badu et al. (2020	42	Healthy	20-72	ICC 0.76	ICC 0.81	ICC 0.84	n/a	n/a

	Li et al. (2018)	11	Healthy		n/a	ICC 0.86	n/a	n/a	n/a
Weighted mean average					ICC 0.70	ICC 0.75	ICC 0.71	n/a	ICC 0.63
M. triceps brachii	0	n/a	n/a	n/a	n/a	n/a	n/a	n/a	n/a
Weighted mean average		n/a	n/a	n/a	n/a	n/a	n/a	n/a	n/a
M. brachioradialis	1	29			29	29	29	n/a	n/a
	Lo et al. (2017)	29	Stroke	30-83	ICC 0.86	ICC 0.90	ICC 0.94	n/a	ICC 0.81
Weighted mean average					ICC 0.86	ICC 0.90	ICC 0.94	n/a	ICC 0.81
M. flexor carpi ulnaris	0	n/a	n/a	n/a	n/a	n/a	n/a	n/a	n/a
Weighted mean average		n/a	n/a	n/a	n/a	n/a	n/a	n/a	n/a
M. extensor carpi radialis brevis	0	n/a	n/a	n/a	n/a	n/a	n/a	n/a	n/a
Weighted mean average		n/a	n/a	n/a	n/a	n/a	n/a	n/a	n/a
M. flexor carpi radialis	0	n/a	n/a	n/a	n/a	n/a	n/a	n/a	n/a
Weighted mean average		n/a	n/a	n/a	n/a	n/a	n/a	n/a	n/a
M. extensor digitorum	0	n/a	n/a	n/a	n/a	n/a	n/a	n/a	n/a
Weighted mean average		n/a	n/a	n/a	n/a	n/a	n/a	n/a	n/a
M. maseter	2	56			16	56	16	16	16
	Yu et al. (2020)	20	Healthy	n/a	n/a	ICC 0.95	n/a	n/a	n/a
	Songv et al. (2021)	20	Stroke	39-57	n/a	ICC 0.99	n/a	n/a	n/a
	Tas et al. (2021)	16	Healthy	18-20	ICC 0.72	ICC 0.76	ICC 0.82	ICC 0.76	ICC 0.82
Weighted mean average					ICC 0.72	ICC 0.91	ICC 0.82	ICC 0.76	ICC 0.82
M. splenius capitis	1	20			20	20	20	n/a	n/a

	Muckelt et al. (2022)	20	Healthy	26-32	ICC 0.80	ICC 0.73	ICC 0.77	n/a	n/a
Weighted mean average					ICC 0.80	ICC 0.73	ICC 0.77	n/a	n/a
M. sternocleido mastoideus	2	38			38	38	38	16	16
	Yeo et al. (2019)	22	post-mastectomy	n/a	ICC 0.65	ICC 0.87	ICC 0.92	n/a	n/a
	Tas et al. (2021)	16	Healthy	18-20	ICC 0.81	ICC 0.78	ICC 0.89	ICC 0.65	ICC 0.73
Weighted mean average					ICC 0.72	ICC 0.83	ICC 0.91	ICC 0.65	ICC 0.73
M. trapezius	3	60			16	60	16	16	16
	Kisilewicz et al. (2018)	24	Neck and shoulder pain		n/a	ICC 0.82	n/a	n/a	n/a
	Liu et al. (2018)	20	Healthy		n/a	ICC 0.97	n/a	n/a	n/a
	Tas et al. (2021)	16	Healthy	18-20	ICC 0.87	ICC 0.79	ICC 0.93	ICC 0.65	ICC 0.50
Weighted mean average					ICC 0.87	ICC 0.86	ICC 0.93	ICC 0.65	ICC 0.50
M. pectoralis major	1	22			n/a	22	n/a	n/a	n/a
	Yeo et al. (2019)	22	post-mastectomy		n/a	ICC 0.34	n/a	n/a	n/a
Weighted mean average					n/a	ICC 0.34	n/a	n/a	n/a
M. extensor cervicale	1	16			16	16	16	16	16
	Tas et al. (2021)	16	Healthy	18-20	ICC 0.93	ICC 0.95	ICC 0.78	ICC 0.91	ICC 0.81
Weighted mean average					ICC 0.93	ICC 0.95	ICC 0.78	ICC 0.91	ICC 0.81
M. erector spinae	2	44			24	44	24	n/a	n/a
	Lohr et al. (2018)	24	Healthy	26-50	ICC 0.94	ICC 0.94	ICC 0.82	n/a	n/a
	Li et al. (2022)	20	Back pain	26-64	n/a	ICC 0.99	n/a	n/a	n/a
Weighted mean average					ICC 0.94	ICC 0.96	ICC 0.82	n/a	n/a
Lumbar extensor muscles	1	80			80	80	80	n/a	n/a
	Wu et al. (2020)	80	Back pain and healthy	50-80	ICC 0.93	ICC 0.95	ICC 0.78	n/a	n/a
Weighted mean average					ICC 0.93	ICC 0.95	ICC 0.78	n/a	n/a
Perineal	1	75			75		75	75	75

	Davidson et al. (2017)	75	Vulvodynia and healthy	15-50	ICC 0.84	n/a	ICC 0.89	ICC 0.91	ICC 0.91
Weighted mean average					ICC 0.84	n/a	ICC 0.89	ICC 0.91	ICC 0.91
Pelvic floor muscle	1	78			78	78	78	78	78
	Rodrigues-de-Souza et al. (2021)	78	urinary incontinence and healthy		ICC 0.86	ICC 0.81	ICC 0.83	ICC 0.76	ICC 0.41
Weighted mean average					ICC 0.86	ICC 0.81	ICC 0.83	ICC 0.76	ICC 0.41
Scar	1	19			19	19	19	19	19
	Gilbert et al. (2020)	19	post-cesarean section	21-40	ICC 0.95	ICC 0.98	ICC 0.91	ICC 0.98	ICC 0.97
Weighted mean average					ICC 0.95	ICC 0.98	ICC 0.91	ICC 0.98	ICC 0.97

2. Intra-rater Reliability

Muscle	Author	N		Alter	Frequency	Stiffness	Decrement	Relaxation	Creep
M. rectus femoris	7	182			142	124	54	13	13
	Lidström et al. (2009)	30	Healthy and Zerepralparese	11	ICC 0.85	n/a	n/a	n/a	n/a
	Mulix et al. (2012)	21	Healthy	20-35	ICC 0.99	ICC 0.99	ICC 0.99	n/a	n/a
	Aird et al. (2012)	20	Healthy	65-82	ICC 0.99	ICC 0.97	ICC 0.99	n/a	n/a
	Lo et al. (2017)	28	Stroke	n/a	ICC 0.81	n/a	n/a	n/a	n/a
	Ko et al. (2018)	13	Spinal cord injuries	n/a	ICC 0.96	ICC 0.98	ICC 0.68	ICC 0.99	ICC 0.98
	Chen et al. (2019)	30	Healthy	23-27	ICC 0.78	ICC 0.80	n/a	n/a	n/a
	Youngjin et al. (2021)	40	Healthy	19-25	n/a	ICC 0.91	n/a	n/a	n/a
Weighted mean average					ICC 0.93	ICC 0.91	ICC 0.92	ICC 0.99	ICC 0.98
M vastus lat.	2	83			30	83	n/a	n/a	n/a
	Chen et al. (2019)	30	Healthy	23-27	ICC 0.74	ICC 0.69	n/a	n/a	n/a
	Bravo-Sanchez et al (2021)	53	Healthy	19-37	n/a	ICC 0.97	n/a	n/a	n/a
Weighted mean average					ICC 0.74	ICC 0.87	n/a	n/a	n/a
M. vastus med.	1	30			30	30	n/a	n/a	n/a
	Chen et al. (2019)	30	Healthy	23-27	ICC 0.80	ICC 0.71	n/a	n/a	n/a

Weighted mean average					ICC 0.80	ICC 0.71	n/a	n/a	n/a
M. biceps femoris	3	74			34	74	34	13	13
	Mulix et al. (2012)	21	Healthy	20-35	ICC 0.99	ICC 0.99	ICC 0.99	n/a	n/a
	Ko et al. (2018)	13	Spinal cord injuries		ICC 0.92	ICC 0.95	ICC 0.82	ICC 0.96	ICC 0.96
	Youngjin et al. (2021)	40	Healthy	19-25	n/a	ICC 0.87	n/a	n/a	n/a
Weighted mean average					ICC 0.96	ICC 0.92	ICC 0.93	ICC 0.96	ICC 0.96
patellar ligament	3	60			60	60	30	n/a	n/a
	Sohirad et al. (2017)	10	Healthy	20-28	ICC 0.96	ICC 0.96	ICC 0.86	n/a	n/a
	Chen et al. (2019)	30	Healthy	23-27	ICC 0.76	ICC 0.80	n/a	n/a	n/a
	Muckelt et al. (2022)	20	Healthy	26-32	ICC 0.52	ICC 0.90	ICC 0.63	n/a	n/a
Weighted mean average					ICC 0.71	ICC 0.86	ICC 0.71	n/a	n/a
M. gastrocnemius med.	5	107			13	107	13	13	13
	Kelly et al. (2018)	30	Healthy		n/a	ICC 0.99	n/a	n/a	n/a
	Ko et al. (2018)	13	Spinal cord injuries		ICC 0.91	ICC 0.93	ICC 0.81	ICC 0.89	ICC 0.89
	Feng et al. (2018)	10	Healthy		n/a	ICC 0.78	n/a	n/a	n/a
	Ge et al. (2021)	14	Spinal cord injuries		n/a	ICC 0.87	n/a	n/a	n/a
	Youngjin et al. (2021)	40	Healthy	19-25	n/a	ICC 0.88	n/a	n/a	n/a
Weighted mean average					ICC 0.91	ICC 0.91	ICC 0.81	ICC 0.89	ICC 0.89
M. gastrocnemius lat.	5	156			13	156	13	13	13
	Leonard et al. (2003)	35	Healthy	22-42	n/a	ICC 0.92	n/a	n/a	n/a
	Ko et al. (2018)	13	Spinal cord injuries	n/a	ICC 0.64	ICC 0.94	ICC 0.79	ICC 0.86	ICC 0.85
	Feng et al. (2018)	10	Healthy	n/a	n/a	ICC 0.93	n/a	n/a	n/a
	Albin et al. (2019)	84	Healthy	21-33	n/a	ICC 0.92	n/a	n/a	n/a
	Ge et al. (2021)	14	Spinal cord injuries	n/a	n/a	ICC 0.91	n/a	n/a	n/a
Weighted mean average					ICC 0.64	ICC 0.9214	ICC 0.79	ICC 0.86	ICC 0.85
M. soleus	1	20			20	20	20	n/a	n/a
	Muckelt et al. (2022)	20	Healthy	26-32	ICC 0.90	ICC 0.66	ICC 0.71	n/a	n/a

Weighted mean average					ICC 0.90	ICC 0.66	ICC 0.71	n/a	n/a
M. tibialis ant.	4	119			79	90	50	n/a	n/a
	Lo et al. (2017)	29	Stroke	30-83	ICC 0.81	n/a	n/a	n/a	n/a
	Youngjin et al. (2021)	40	Healthy	19-25		ICC 0.89	n/a	n/a	n/a
	Muckelt et al. (2022)	20	Healthy	26-32	ICC 0.80	ICC 0.85	ICC 0.95	n/a	n/a
	Agoriwo et al. (2022)	30	Parkinson	n/a	ICC 0.94	ICC 0.97	ICC 0.95	n/a	n/a
Weighted mean average					ICC 0.86	ICC 0.91	ICC 0.95	n/a	n/a
Achilles tendon	8	157			43	157	43	13	13
	Sohirad et al. (2017)	10	Healthy	20-28	ICC 0.96	ICC 0.96	ICC 0.94	n/a	n/a
	Ko et al. (2018)	13	Spinal cord injuries		ICC 0.82	ICC 0.83	ICC 0.58	ICC 0.82	ICC 0.89
	Long Liu et al. (2018)	20	Healthy	22-28	n/a	ICC 0.89	n/a	n/a	n/a
	Feng et al. (2018)	10	Healthy		n/a	ICC 0.86	n/a	n/a	n/a
	Schneebeli et al. (2020)	40	Healthy		n/a	ICC 0.93	n/a	n/a	n/a
	Ge et al. (2021)	14	Spinal cord injuries		n/a	ICC 0.89	n/a	n/a	n/a
	Chang et al. (2020)	30	Healthy		n/a	ICC 0.92	n/a	n/a	n/a
	Muckelt et al. (2022)	20	Healthy	26-32	ICC 0.52	ICC 0.9	ICC 0.63	n/a	n/a
Weighted mean average					ICC 0.71	ICC 0.91	ICC 0.69	ICC 0.82	ICC 0.89
Plantar fascia	1	20			20	20	20	n/a	n/a
	Muckelt et al. (2022)	20	Healthy	26-32	ICC 0.85	ICC 0.96	ICC 0.55	n/a	n/a
Weighted mean average					ICC 0.85	ICC 0.96	ICC 0.55	n/a	n/a

Muscle	Author	N		Alter	Frequency	Stiffness	Decrement	Relaxation	Creep
M. infraspinatus	2	65			35	65	35	n/a	n/a
	Kelly et al. (2018)	30	Healthy	n/a	n/a	ICC 0.98	n/a	n/a	n/a
	Roch et al. (2020)	35	Shoulder pain	42	ICC 0.97	ICC 0.96	ICC 0.96	n/a	n/a
Weighted mean average					ICC 0.97	ICC 0.97	ICC 0.96	n/a	n/a
M. deltoideus	2	81			81	81	81	n/a	n/a

	Muckelt et al. (2022)	20	Healthy	26-32	ICC 0.75	ICC 0.92	ICC 0.94	n/a	n/a
	Chuang et al. (2012)	61	Stroke	44-66	ICC 0.92	ICC 0.93	ICC 0.86	n/a	n/a
Weighted mean average					ICC 0.88	ICC 0.93	ICC 0.88	n/a	n/a
M. biceps brachii	8	328			255	300	227		70
	Leonard et al. (2003)	35	Healthy	22-42	n/a	ICC 0.92	n/a	n/a	n/a
	Chuang et al. (2012)	12	Stroke	40-62	ICC 0.84	ICC 0.89	ICC 0.90	n/a	n/a
	Chuang et al. (2012)	61	Stroke	44-66	ICC 0.94	ICC 0.92	ICC 0.93	n/a	n/a
	Lo et al. (2017)	28	Stroke	n/a	ICC 0.75	n/a	n/a	n/a	n/a
	Drenth et al. (2017)	70	Paratonia	n/a	ICC 0.60	ICC 0.59	ICC 0.71	n/a	ICC 0.54
	Maruwsiak et al. (2018)	38	Healthy and Parkinson	73-81	n/a	ICC 0.98	n/a	n/a	n/a
	Van Deun et al. (2018)	54	Healthy and Paratonia	24-98	ICC 0.83	ICC 0.91	ICC 0.93	n/a	n/a
	Agoriwo et al. (2022)	30	Parkinson	n/a	ICC 0.94	ICC 0.97	ICC 0.9	n/a	n/a
Weighted mean average					ICC 0.80	ICC 0.85	ICC 0.86	n/a	ICC 0.54
M. triceps brachii	3	111			73	111	73		
	Chuang et al. (2012)	12	Stroke	40-62	ICC 0.95	ICC 0.96	ICC 0.93	n/a	n/a
	Chuang et al. (2012)	61	Stroke	44-66	ICC 0.87	ICC 0.88	ICC 0.89	n/a	n/a
	Maruwsiak et al. (2018)	38	Healthy and Parkinson	73-81	n/a	ICC 0.97	n/a	n/a	n/a
Weighted mean average					ICC 0.88	ICC 0.92	ICC 0.90	n/a	n/a
M. brachioradialis	2	45			45	17	17	n/a	n/a
	Jarocka et al. (2007)	17	Healthy	20-22	ICC 0.96	ICC 0.92	ICC 0.97	n/a	n/a
	Lo et al. (2017)	28	Stroke	n/a	ICC 0.82	n/a	n/a	n/a	n/a
Weighted mean average					ICC 0.87	ICC 0.92	ICC 0.97	n/a	n/a
M. flexor carpi ulnaris	3	198			198	198	198	n/a	n/a
	Chuang et al. (2012)	67	Stroke	50-76	ICC 0.91	ICC 0.89	ICC 0.90	n/a	n/a

	Chuang et al. (2012)	61	Stroke	44-66	ICC 0.93	ICC 0.92	ICC 0.89	n/a	n/a
	Saldiran et al. (2022)	70	Healthy	18-35	ICC 0.92	ICC 0.86	ICC 0.82	n/a	n/a
Weighted mean average					ICC 0.92	ICC 0.89	ICC 0.87	n/a	n/a
M. extensor carpi radialis brevis	1	70			70	70	70	n/a	n/a
	Saldiran et al. (2022)	70	Healthy	18-35	ICC 0.98	ICC 0.92	ICC 0.56	n/a	n/a
Weighted mean average					ICC 0.98	ICC 0.92	ICC 0.56	n/a	n/a
M. flexor carpi radialis	3	158			158	158	158	n/a	n/a
	Chuang et al. (2012)	67	Stroke	50-76	ICC 0.95	ICC 0.93	ICC 0.93	n/a	n/a
	Chuang et al. (2012)	61	Stroke	44-66	ICC 0.95	ICC 0.94	ICC 0.92	n/a	n/a
	Agoriwo et al. (2022)	30	Parkinson	n/a	ICC 0.83	ICC 0.86	ICC 0.85	n/a	n/a
Weighted mean average					ICC 0.93	ICC 0.92	ICC 0.91	n/a	n/a
M. extensor digitorum	2	128			128	128	128	n/a	n/a
	Chuang et al. (2012)	67	Stroke	50-76	ICC 0.90	ICC 0.89	ICC 0.81	n/a	n/a
	Chuang et al. (2012)	61	Stroke	44-66	ICC 0.92	ICC 0.92	ICC 0.83	n/a	n/a
Weighted mean average					ICC 0.91	ICC 0.90	ICC 0.82	n/a	n/a
M. maseter	2	56			16	56	16	16	16
	Yu et al. (2020)	20	Healthy	n/a	n/a	ICC 0.78	n/a	n/a	n/a
	Songv et al. (2021)	20	Stroke	39-57	n/a	ICC 0.99	n/a	n/a	n/a
	Tas et al. (2021)	16	Healthy	18-20	ICC 0.76	ICC 0.86	ICC 0.88	ICC 0.66	ICC 0.71
Weighted mean average					ICC 0.76	ICC 0.88	ICC 0.88	ICC 0.66	ICC 0.71
M. splenius capitis	1	20			20	20	20		
	Muckelt et al. (2022)	20	Healthy	26-32	ICC 0.75	ICC 0.57	ICC 0.94	n/a	n/a

Weighted mean average					ICC 0.75	ICC 0.57	ICC 0.94	n/a	n/a
M. sternocleidomastoideus	2	38			38	38	38	16	16
	Yeo et al. (2019)	22	post-mastectomy		ICC 0.87	ICC 0.93	ICC 0.94	n/a	n/a
	Tas et al. (2021)	16	Healthy		ICC 0.74	ICC 0.85	ICC 0.86	ICC 0.74	ICC 0.86
Weighted mean average					ICC 0.82	ICC 0.90	ICC 0.91	ICC 0.74	ICC 0.86
M. trapezius	3	60			16	60	16	16	16
	Kisilewicz et al. (2018)	24	Neck and shoulder pain	n/a	n/a	ICC 0.87	n/a	n/a	n/a
	Liu et al (2018)	20	Healthy	n/a	n/a	ICC 0.97	n/a	n/a	n/a
	Tas et al. (2021)	16	Healthy	18-20	ICC 0.81	ICC 0.82	ICC 0.76	ICC 0.74	ICC 0.52
Weighted mean average					ICC 0.81	ICC 0.89	ICC 0.76	ICC 0.74	ICC 0.52
M. pectoralis major	1	22			n/a	22	n/a	n/a	n/a
	Yeo et al. (2019)	22	post-mastectomy	n/a	n/a	ICC 0.85	n/a	n/a	n/a
Weighted mean average					n/a	ICC 0.85	n/a	n/a	n/a
M. extensor cervicale	1	16			16	16	16	16	16
	Tas et al. (2021)	16	Healthy	18-20	ICC 0.67	ICC 0.77	ICC 0.82	ICC 0.85	ICC 0.79
Weighted mean average					ICC 0.67	ICC 0.77	ICC 0.82	ICC 0.85	ICC 0.79
M. erector spinae	3	74			24	74	24	n/a	n/a
	Kelly et al. (2018)	30	Healthy	n/a	n/a	ICC 0.99	n/a	n/a	n/a
	Lohr et al. (2018)	24	Healthy	26-50	ICC 0.98	ICC 0.97	ICC 0.93	n/a	n/a
	Li et al. (2022)	20	Back pain	26-64	n/a	ICC 0.90	n/a	n/a	n/a
Weighted mean average					ICC 0.98	ICC 0.96	ICC 0.93	n/a	n/a
Lumbar extensor muscles	0	n/a	n/a	n/a	n/a	n/a	n/a	n/a	n/a
Weighted mean average		n/a	n/a	n/a	n/a	n/a	n/a	n/a	n/a
Perineal	1	75			75		75	75	75

	Davidson et al. (2017)	75	Vulvodynia and healthy	15-50	ICC 0.88	n/a	ICC 0.91	ICC 0.92	ICC 0.90
Weighted mean average					ICC 0.88	n/a	ICC 0.91	ICC 0.92	ICC 0.90
Pelvic floor muscle	1	78			78	78	78	78	78
	Rodrigues-de-Souza et al. (2021)	78	urinary incontinence and healthy	n/a	ICC 0.85	ICC 0.83	ICC 0.77	ICC 0.82	ICC 0.74
Weighted mean average					ICC 0.85	ICC 0.83	ICC 0.77	ICC 0.82	ICC 0.74
Scar	1	19			19	19	19	19	19
	Gilbert et al. (2020)	19	post-cesarean section	21-40	ICC 0.99	ICC 0.99	ICC 0.99	ICC 0.99	ICC 0.99
Weighted mean average					ICC 0.99	ICC 0.99	ICC 0.99	ICC 0.99	ICC 0.99
